# The Functional Foundations of Episodic Memory Remain Stable Throughout the Lifespan

**DOI:** 10.1093/cercor/bhaa348

**Published:** 2020-11-30

**Authors:** Didac Vidal-Piñeiro, Markus H Sneve, Inge K Amlien, Håkon Grydeland, Athanasia M Mowinckel, James M Roe, Øystein Sørensen, Lars H Nyberg, Kristine B Walhovd, Anders M Fjell

**Affiliations:** 1 Department of Psychology, Centre for Lifespan Changes in Brain and Cognition, University of Oslo, Oslo 0317, Norway; 2 Umeå Centre for Functional Brain Imaging, S-90187 Umeå, Sweden; 3 Physiology Section, Department of Integrative Medical Biology, Umeå University, S-90187 Umeå, Sweden; 4 Department of Radiation Sciences, Diagnostic Radiology, Umeå University, S-90187 Umeå, Sweden; 5 Department of Radiology and Nuclear Medicine, Oslo University Hospital, 04024 Oslo, Norway

**Keywords:** aging, development, encoding, fMRI, neuroimaging

## Abstract

It has been suggested that specific forms of cognition in older age rely largely on late-life specific mechanisms. Here instead, we tested using task-fMRI (*n* = 540, age 6–82 years) whether the functional foundations of successful episodic memory encoding adhere to a principle of lifespan continuity, shaped by developmental, structural, and evolutionary influences. We clustered regions of the cerebral cortex according to the shape of the lifespan trajectory of memory activity in each region so that regions showing the same pattern were clustered together. The results revealed that lifespan trajectories of memory encoding function showed a continuity through life but no evidence of age-specific mechanisms such as compensatory patterns. Encoding activity was related to general cognitive abilities and variations of grey matter as captured by a multi-modal independent component analysis, variables reflecting core aspects of cognitive and structural change throughout the lifespan. Furthermore, memory encoding activity aligned to fundamental aspects of brain organization, such as large-scale connectivity and evolutionary cortical expansion gradients. Altogether, we provide novel support for a perspective on memory aging in which maintenance and decay of episodic memory in older age needs to be understood from a comprehensive life-long perspective rather than as a late-life phenomenon only.

## Introduction

Over the last few years, research has demonstrated that the structural foundations of general cognitive abilities are largely constant throughout life (Karama et al. 2014; Walhovd et al. 2016), being embedded into fundamental aspects of brain organization as captured by evolutionary expansion patterns or connectivity gradients (Margulies et al. 2016; Sneve et al. 2019). However, it is unknown whether the functional foundations supporting specific forms of cognition are equally stable or rather dynamic through life. In supporting signature aspects of human cognition like autonoetic consciousness and future thinking (Tulving 1983; Schacter et al. 2012), episodic memory represents a crucial ability in everyday function. Its vulnerability, particularly in old age, has thus attracted much research effort, leading researchers to postulate distinct age-specific mechanisms to explain brain-behavior correlates at different periods in life (Cabeza et al. 2018; Stern et al. 2020). Yet, lifespan researchers have emphasized integrative accounts of lifelong changes in cognitive abilities—and episodic memory in particular—in which development and decay of brain structure often represent a key fundament for brain function and cognitive change (Schulz and Heckhausen 1996; Craik and Bialystok 2006; Shing et al. 2010; Nyberg et al. 2012; Walhovd et al. 2018). Using a novel and multifaceted analytic approach, we looked for evidence of continuous and age-specific functional mechanisms supporting episodic memory and how these are related to fundamental variations in brain structure and cognition throughout the lifespan. As the main goal, we aimed to assess whether episodic memory function represents fundamental aspects of life-long brain organization and continuity, similar to what has been established for general cognitive abilities.

**Figure 1:**
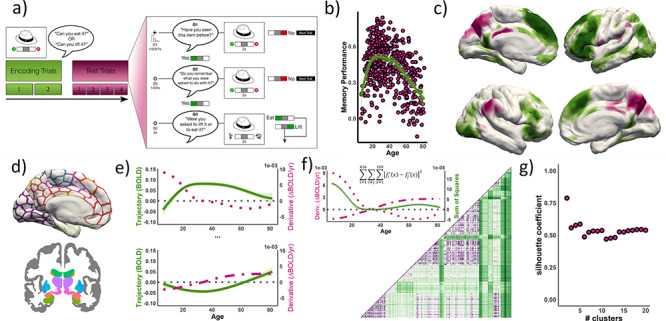
Experimental scheme. (*a*) Experimental design; adapted from Roe et al. (2020). (*b*) Source memory performance across the lifespan sample (proportional, corrected for guessing). (*c*) Subsequent memory contrast (BOLD_S > i_: source vs. item memory encoding) for the entire sample (*n* = 540). See Supplementary Methods and Results. Green and purple regions denote positive and negative subsequent memory effects, respectively. (*d*–*g*) Lifespan Parcellation pipeline. Brain clusters characterized by different ‘canonical’ trajectories were established from subsequent source memory encoding effects across life. (*d*) 416 ROIs were selected corresponding to the “Schaefer” (Schaefer Local–Global parcellation) and “fsaseg” cortical and subcortical atlases. (*e*) For each ROI, we fitted a lifespan trajectory to encoding activity and computed its derivative. (*f*) A dissimilarity matrix, based on pairwise distance of derivatives, was computed to group regions with similar age trajectories. The matrix was based on a least-squared sum (416 ROIs; 100 equispaced age points). (*g*) The similarity matrix was fed into a *k*-medoids algorithm and the optimal partition (*k* = 5) was established by the silhouette width coefficients.

Despite ample evidence of age differences in associative encoding function, lifespan approaches are still rare (c.f. Shing et al. 2016). Yet, there are recurrent findings shared by both child development and aging research. Common findings include decreased activity in the prefrontal cortex and default-network regions; the latter typified as reduced activity associated with memory failure; that is, “negative” memory effects (Miller et al. 2008; Güler and Thomas 2012; Shing et al. 2016; Amlien et al. 2018; Vidal-Piñeiro et al. 2019). Age-related changes of encoding activity in the medial temporal lobe and in posterior perceptual regions—except for the dorsal visual pathway—are less clear (see Shing et al. 2010; Wang and Cabeza 2016 for reviews). Age-related changes in neural function are thought to mediate gains and losses of memory performance through life. The brain organization is constrained by ontogenic development and determined by several factors such as the distance from primary sensorimotor regions or the genetic profiles. Cognition is embedded into such aspects of brain organization as are a region’s flexibility, maturation, and vulnerability profiles (Goldman-Rakic 1988; Mesulam 1998). An alternative view suggests old age individuals invoke additional mechanisms to maintain cognitive performance. These mechanisms appear as a “reaction” to the deleterious effects of age, brain pathology, or other brain insults and may involve over-recruitment of existing networks, recruitment of new networks due to reorganization or strategy selection, and more efficient use of existing resources (Cabeza et al. 2018; Stern et al. 2020). These age-specific mechanisms are either unavailable or unused in young adulthood.

Here, we delineated the lifespan trajectories associated with episodic encoding success, using fMRI data from 540 healthy individuals from 6 to 82 years during an incidental source-item encoding task (Fig. 1*a*–*c*). Region-wise encoding activity throughout the entire brain was fit to age using generalized additive models (GAM) and the most characteristic lifespan trajectories were established based on a clustering approach (Fig. 1*d*–*g*). Encoding was indexed using a source versus item memory contrast that isolates the binding aspects of episodic memory and is highly sensitive to age. We reasoned that support for an account of lifelong continuity in the brain foundations of episodic encoding would require that: 1) The lifespan trajectories of encoding activity cluster into known networks with a meaningful function and topology. We expected the lifespan clustering procedure to reveal different systems that are characterized by specific maturation and vulnerability profiles. 2) The clusters of activity trajectories are embedded in fundamental aspects of brain organization as quantified by the degree of cortical expansion through evolution, the capacity for integration of information, and the principal functional gradient which reflects the differentiation between unimodal and transmodal cortex (Van Essen and Dierker 2007; Yeo et al. 2015; Margulies et al. 2016). The clusters more affected by age should be characterized by aspects of brain organization that permit more flexible, unconstrained computations. 3) The trajectories are not determined by age-specific profiles of activity at middle or older age, that is, defined by increased/decreased activity in late-life only 4) Interindividual variations of activity in developmentally sensitive clusters are related to core cognitive abilities and structural features that govern brain and cognitive variability in childhood and older age. Evidence for age-specific mechanisms in older age would require one of the following 1) trajectories defined by lack of activity during childhood and young adulthood and increased activity in middle or older age, 2) trajectories defined by higher activity in older versus younger age or 3) trajectories characterized by less activity in old adulthood and a negative activity-performance relationship.

## Material and Methods

### Participants

The final sample included 540 individuals (females = 366, age = 39.1 [SD = 18.5] years, age range = 6–82 years). The study was approved by the Regional Ethical Committee of South Norway. All participants ≥12 years gave written informed consent, all participants <12 years gave oral informed consent and, for all participants <18 years, written informed consent was obtained from their legal guardians. All participants were screened through health and neuropsychological interviews. See Supplementary Methods for additional sample and exclusion criteria details. Participants’ data were discarded due to technical errors, faulty acquisitions or a low number of trials in a condition of interest (<6 trials; *n* = 14).

### Experimental Design and Behavioral Analysis

The experiment consisted of an incidental encoding task and a memory test after approximately 90 min. Both tasks took place inside the scanner. Only the fMRI data for the encoding session is used in the current study. The experimental design is thoroughly described elsewhere (Sneve et al. 2015; Vidal-Piñeiro et al. 2019). See Figure 1*a* and Supplementary Methods for further details.

Briefly, the encoding and the retrieval tasks consisted of two and four runs, respectively, that included 50 trials each. All runs started and ended with an 11 s baseline recording period in which a central fixation cross was present. An additional baseline period was also presented once in the middle of each run. The trials started with a voice asking either “Can you eat it? “or “Can you lift it?” (in Norwegian) (25 times each). After 1 s, a picture of an item appeared on the screen together with a “Yes/No” response indicator. Note that responses contained an objective component and thus were neither correct nor incorrect (e.g., Can you lift a crocodile?). Participants were instructed to link the item with the action self-referentially. The subject had 2 s to produce a response before the object was replaced by a central fixation cross (intertrial interval [ITI] = 2.98 [SD 2.49] s; range = 1–7 s [exponential distribution over four discrete it is]. During retrieval, test trials started with the following question (Q1): “Have you seen this item before”. Then, a picture of an item appeared, and the participant was instructed to indicate “Yes or No”. In each run, 25 old and 25 new items were presented in a pseudorandomized order. Each object stayed on the screen for 2 s; if the participant responded that the item was new or did not respond, the trial ended. If the participant remembered seeing the item, a new question followed (Q2): “Can you remember what you were supposed to do with the item?”. A “No” response ended the trial, whereas a “Yes” response was followed by a final question (Q3): “Were you supposed to eat it or lift it?”. Here, the participant had to choose between the two actions “Eat” or “Lift” associated with the item encoding (“I imaged eating/lifting the item during the encoding phase”).

For behavioral analysis, test trial responses to old items were classified as follows: source memory (Yes response to Q1 and Q2 and correct response to Q3); item memory (correct Yes response to Q1 and either a No response to Q2, or incorrect response to Q3); or miss (incorrect No response to Q1). Memory performance in the task was assessed with a corrected source memory performance index (source memory—incorrect source memories [Yes response to Q1 and Q2 and correct response to Q3]). This correction tentatively accounts for the probability of correct source memories at chance (0.5 given Yes response to Q1 and Q2) and controls for processes such as false memories, threshold criteria in Q2 or guessing behavior that affects the raw estimates of source memory performance (Vidal-Piñeiro et al. 2017, 2019). The correlation between the corrected and the uncorrected source memory index was *r* = 0.93.

### MRI Acquisition and Preprocessing

Imaging data were collected using a 20-channel head coil on a 3T MRI (Skyra, Siemens Medical Solutions, Ge) at Rikshospitalet (Oslo). Each encoding run consisted of 134 volumes with the following functional imaging parameters: 43 transversally oriented slices were measured using a BOLD-sensitive T2*-weighted EPI sequence (TR = 2390 ms, TE = 30 ms, flip angle = 90°; voxel size = 3 × 3 × 3 mm; FOV = 224 × 224 mm; interleaved acquisition; generalized autocalibrating partially parallel acquisitions acceleration [GRAPPA] factor = 2). Three dummy volumes were collected at the start of each fMRI run to avoid T1 saturation effects in the analyzed data. Anatomical T1-weighted (T1w) magnetization-prepared rapid gradient echo (MPRAGE) images consisted of 176 sagittally oriented slices and were obtained using the following turbo field echo pulse sequence: TR = 2300 ms, TE = 2.98 ms, flip angle = 8°, voxel size = 1 × 1 × 1 mm, FOV = 256 × 256 mm. Additionally, a standard double-echo gradient-echo field map sequence was acquired for distortion correction of the echo-planar images. Visual stimuli were displayed in the scanner with an NNL 32-inch LCD monitor (NordicNeuroLab). Participants responded using the ResponseGrip system (NordicNeuroLab).

The MRI dataset was converted to Brain Imaging Data Structure format (BIDS) (Gorgolewski et al. 2016) while cortical reconstruction and volumetric segmentation of the T1-weighted scans were performed with the FreeSurfer v.6.0 pipeline (http://surfer.nmr.mgh.harvard.edu/fswiki) (Fischl and Dale 2000). The fMRI analyses were constrained to a set of 416 ROIs covering the entire cortical and subcortical space. Subcortical ROIs regions were defined in native space based on the FreeSurfer automatic subcortical segmentation “aseg” with the additional division of the hippocampi along the anterior–posterior axis (Poppenk et al. 2013). Cerebellum was not included due to partial acquisition for this region in several participants. For the cortical surface, 200 ROIs were defined per hemisphere on each participants’ native reconstructed surface based on the Local–Global Parcellation of the Human Cerebral Cortex (Fig. 1*d*; Schaefer et al. 2018; Schaefer Local-Global parcellation).

fMRI data were processed using the “fMRIPrep” preprocessing pipeline (Esteban et al. 2019). See Supplementary Methods for a detailed description. The pipeline included skull-stripping, susceptibility distortions correction, motion correction, co-registration with the anatomical reference using boundary-based registration, and slice-timing correction. Post-“fMRIPrep” nuisance regression removed effects of estimated motion confounds (3 translations, 3 rotations, framewise displacement), and six “aCompCor” principal components derived from an eroded WM/CSF-mask. Data were high-pass filtered (128 s cut-off) using a discrete cosine filter. Volume resampling was performed in a single interpolation step and was then sampled to each participants’ cortical surface space.

### fMRI Analysis

First-level general linear models (GLM) were carried out with FSFAST (https://surfer.nmr.mgh.harvard.edu/fswiki/FsFast). For each participant and encoding run, we set up a first-level GLM consisting of the conditions of interest, with onsets and durations corresponding to the experimental trial period (i.e., 2 s epochs that comprised the entire period of picture presentation—and hence the response time-window—as well as their temporal derivatives. The temporal derivatives were included for first-level analyses purposes and orthogonalized (Pernet 2014). The contrasts were used to account for any age-related hemodynamic changes that could confound the results. GLMs were estimated both in the cortical surfaces and in the subcortical structures of interest in each subject’s native space. Events were assigned to a given condition based on the participant’s response during the subsequent memory test. The regressors were convolved with a double-gamma canonical hemodynamic response function (HRF). The conditions of interest were source and item memory conditions as defined in the behavioral analysis based on the subsequent memory judgments (Source = subsequent item-source association [Yes response to Q1 and Q2 and correct response to Q3]; Item = subsequent item memory without memory for the association [correct Yes response to Q1 and either a No response to Q2, or incorrect response to Q3]). Two additional regressors were included to soak up BOLD variance associated with miss memory trials and with trials with no response. Finally, for each participant, percent signal change during source and item memory encoding was estimated and contrasted to produce estimates of episodic memory encoding, which were then averaged over voxels/vertices within ROIs. All between-subjects analyses were performed in R-environment (https://www.r-project.org/; v.3.5.2). Significance values were corrected using false discovery rate (pFDR) as implemented by Benjamini and Yekutieli (2001) which controls for positive dependency amongst variables across all families of tests. We used “ggplot2”, “ggseg”, and “freesurfer” software for visualization (Wickham 2016; Mowinckel and Vidal-Piñeiro 2019).

### Clustering of Lifespan Trajectories of Encoding Activity

We used a clustering approach to group the different regions according to their lifespan trajectories of encoding activity so that regions with a similar age profile would be assigned to the same cluster (i.e., inverted-U shape vs. monotonic change). See clustering pipeline in Figure 1*d*–*g*. Brain clusters characterized by different ‘canonical’ trajectories were established from subsequent source memory encoding effects across life. In each ROI (*n* = 416), we fitted age on the episodic memory contrast using GAM models as implemented in the “vows” package (Reiss et al. 2014). GAM is a flexible, nonparametric fitting routine with relaxed assumptions about the relationship between variables (Wood 2017). The technique is capable of fitting nonlinear relationships through local smoothing effects, is independent of any predefined model, and robust to selection range (i.e., age range) and non-normally distributed variables (Fjell et al. 2013). In each GAM, we fitted the activity values using age as the smoothing term (knots = 10) and sex as a covariate. The GAM models were re-run after excluding outlier values, defined as observations where residuals were >4 SD above or below the fitting (1.39 outliers were removed per model). For each fitting, we saved mean activity (Intercept), age effects, and edf (estimated degrees of freedom). Next, we computed the derivative of each lifespan trajectory based on a numerical approximation with the “numDeriv” package (Fig. 1*e*; Gilbert and Varadhan 2019). To group regions with similar age trajectories of encoding function, we obtained a dissimilarity matrix by computing the distance between each pair of ROIs’ derivatives—based on a least square sum (Fig. 1*f*; we draw 100 equispaced samples along the age-range continuum). We used the derivatives instead of the “raw” trajectories as the former removes the effects of the intercept (mean activity). Pairwise comparison of “raw” trajectories would lead to cluster solutions based to a large degree on the intercept as age tends to exert a modulatory (and thus, comparatively minor) influence on activity. Regions without evidence of subsequent memory activity (*n* = 74) were removed from the dissimilarity matrix. Regions showing subsequent memory activity were fed into a *k*-medoids algorithm and the optimal partition (*k* = 5; *k* representing the number of different clusters) was established by the silhouette width coefficients (Fig. 1*g*). The average silhouette coefficient estimates how well, on average, each object lies withing its cluster and is regarded as a proxy of clustering quality. See Supplementary Figure 1 for a solution at *k* = 8, an alternative solution based on the silhouette coefficients. To test the stability of the cluster solution, we repeated the analysis using half-split replication (Supplementary Fig. 2). For both half-split samples, an optimal partition was found at *k* = 5 based on the silhouette width coefficients; one half-split sample also had *k* = 7 as an alternative solution. A GAM analysis showed that in-scanner motion, quantified as mean DVARS, was associated with age (*F* = 7.7, *P* < 0.001, edf = 6.0) exhibiting a U-shape trajectory with a steeper slope during childhood and adolescence than in older age). To explore possible effects of motion to the clustering solution, we repeated the clustering analysis after the removal of 10% of the participants with higher mean DVARS (Supplementary Fig. 3).

### Relationship between Encoding Activity, Cognition, and Grey Matter Variation

For each cluster, the first PCA component across ROIs was used as a participant’s episodic encoding measure. Cognition was assessed using: memory performance in the task, California Verbal Learning Test (CVLT) total learning score, and vocabulary and matrix reasoning raw scores. CVLT total learning score is used as a measure of episodic memory encoding capacity. Matrices and vocabulary tests were used as indices for fluid and crystallized abilities. We considered both factors of interest in line with existing frameworks for cognitive change throughout the lifespan (Baltes et al. 2006; Craik and Bialystok 2006). We used a linked Independent Component Analysis (ICA; Groves et al. 2011, 2012) to derive modes of grey matter (GM) variation using three different modalities: cortical thickness and area based on cortical surface reconstructions (Fischl and Dale 2000), and volume from a voxel-based morphometry (VBM) protocol (Good et al. 2001). The modes of GM variation (*n* = 70 components) were obtained as implemented by FLICA and following the pipeline described in Douaud et al. (2014) (http://fsl.fmrib.ox.ac.uk/fsl/fslwiki/FLICA; Supplementary Methods and Results). We then selected those components which showed a practical significance with age (*r*^2^ > 0.15). For each cluster, we fitted activity using GAM models with age and cognition/GM variation as smoothing terms and sex as a covariate. Note that age is also a covariate; age was modeled as a smoothed term to capture non-linear relationships between age and activity. Note also that these GAM analyses were run as implemented in the “mgcv” package (Wood 2017) as it allows for multiple smoothing terms and user-defined penalties to curve wiggliness (gamma = 2).

### Relationship between Regional Solution and Topological Organization

We assessed the topological relationship between cluster assignment and fundamental aspects of brain organization, namely the first component of functional connectivity (Margulies et al. 2016), flexibility (Yeo et al. 2015), and macaque-human cortical expansion maps (Van Essen and Dierker 2007; Hill et al. 2010). Flexibility quantifies the capacity of a region to support multiple tasks and thus to integrate specialized brain networks (Yeo et al. 2015). Macaque-human cortical expansion maps estimate the expansion of the cortical surface throughout primate evolution. Amongst others, this measure relates to the hierarchical organization of the human cortex, the dendritic and synaptic architecture, and the degree of postnatal expansion (Hill et al. 2010; Buckner and Krienen 2013). The first component of functional connectivity closely reflects the distance from primary sensory and motor areas and is a good proxy of a representational hierarchy (Margulies et al. 2016). All maps were available as open resources. For each map, values were averaged within ROIs. Surface maps were used for cortical data. Subcortical information—in volume-based format—was only available for the flexibility index. We ran a right-to-left registration for the evolutionary expansion map as this was available only for the right hemisphere. For the evolutionary expansion map, we used ranked-values due to the exponential distribution of evolutionary expansion. Permutation testing was used to establish the significance of topological relationships (*n* = 10 000 permutations). We generated a random distribution by randomly assigning ROIs to clusters. Only differences amongst clusters greater than those established by the random distribution (FDR-corrected) were considered significant.

## Results

### Delineation of Lifespan Trajectories of Encoding Activity

As expected, age was related to memory performance (*F* = 37.4, *P* < 0.001, edf [estimated degrees of freedom; index of curve complexity] = 4.5; Fig. 1*c*), revealing an inversed U-shape lifespan trajectory. See complete behavioral results in Supplementary Figure 4 and Supplementary Table 1.

**Figure 2:**
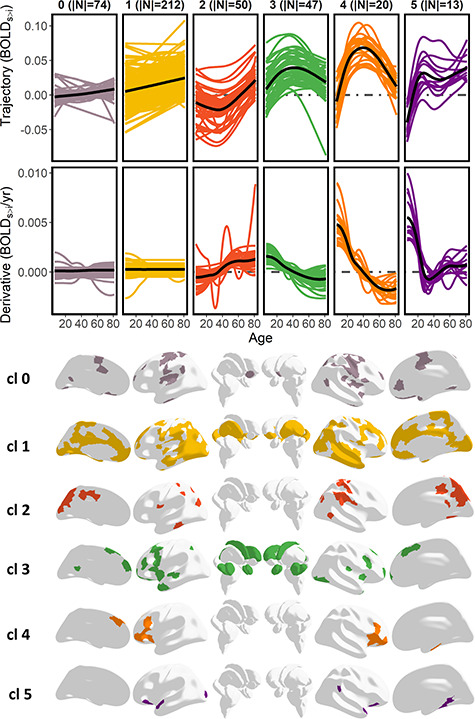
Cluster solution based on the derivatives of the lifespan trajectories of encoding activity. Upper panel: Lifespan trajectories and the derivatives of encoding activity grouped by cluster. Lower panel: ROI assignment by cluster. BOLD_S > i_ = Subsequent source versus Item memory fMRI contrast. Note that cluster 0 was defined prior to the clustering analysis as regions not showing subsequent memory effects. (|*N*| = number of ROIs in a cluster).

**Figure 3:**
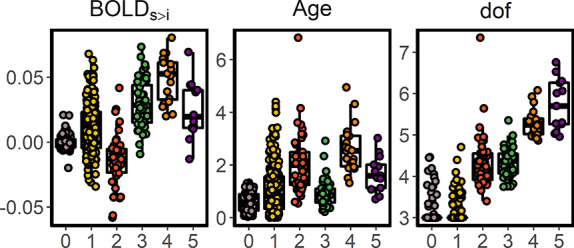
Mean BOLD activity for the source-v-item memory contrast (BOLD_S > I_), age effects (−log10(*p*)), and estimated degrees of freedom (range = 3–9) for each ROI grouped by cluster.

We reduced the number of regions in a data-driven manner by clustering the brain based on the pairwise similarity of the lifespan trajectories (derivatives) of episodic encoding function. We used a k-means clustering algorithm, which yielded an optimal partition at *k* = 5 with an average silhouette coefficient of 0.59. Figure 2 displays the resulting 5-partition arrangement of cortical and subcortical regions based on the canonical trajectories of encoding activity during the lifespan. For each cluster, a canonical trajectory refers to the mean lifespan trajectory against which the other trajectories are compared and adhere to. See in Figure 3 the effects of age, edf, and mean activity per ROI grouped by cluster; see stats in Supplementary Appendix 1. Note that by using a distance matrix based on the derivatives, we partitioned the brain solely from the lifespan trajectories, disregarding the intercept (i.e., mean activity). Thus, regions are grouped together if their episodic encoding activity shows the same age-relationships (e.g., two regions with similar lifelong trajectories will group together regardless of whether they show positive or negative memory effects).

An initial group of regions (cluster 0) was obtained prior to the partition algorithm (|*N*| = 74) (|*N*| = number of ROIs in a cluster), which included regions that did not show evidence of activity associated with encoding success throughout the lifespan. Most other regions were assigned to cluster 1 (|*N*| = 212), which encompassed large parts of cortex and subcortex including the posterior hippocampi. The trajectories of cluster 1 tended to exhibit weak monotonic increments of activity throughout the lifespan. Cluster 2 consisted of |*N*| = 50 regions, which were located almost entirely in the posteromedial and in the inferior parietal lateral cortices. Cluster 2 showed a canonical U-shape lifespan trajectory and consisted of regions that showed negative memory effects during young adulthood. Cluster 3 consisted of |*N*| = 47 regions from bilateral prefrontal and subcortical regions, including the anterior hippocampi, that mapped onto a weak inverted-U shape trajectory. Most of the regions exhibited positive subsequent memory effects during young and middle adulthood. Most of the |*N*| = 20 regions of cluster 4 mapped to the inferior frontal gyrus, bilaterally, extending also to the left superior frontal cortex and the right parahippocampal gyrus. Episodic encoding activity in cluster 4 showed a steep inverted U-shaped trajectory over the lifespan. All these regions exhibited positive subsequent memory effects during young and middle adulthood and were significantly related to age (all ROIs *P* < 0.05). Finally, cluster 5 consisted of |*N*| = 13 regions and exhibited a canonical childhood and adolescent “developmental” trajectory with activity increasing in childhood before reaching a plateau that lasted throughout adulthood. Cluster 5 included anterior temporal, pars orbitalis regions bilaterally as well as parts of the right temporoparietal and parahippocampal cortices. Overall, the results showed a continuity of the canonical trajectories throughout the lifespan as 1) patterns of activity developed and decayed at younger and older age, respectively (clusters 2–4); 2) developed at younger age and later stabilized (cluster 5) or, 3) showed a monotonical pattern through the entire life (cluster 1). Critically, none of the trajectories exhibited late-life profiles of activity with distinct patterns emerging at middle or older age. A half-split sample replication showed the solution was largely stable (Supplementary Fig. 2). The clustering solution remained stable after the removal of participants with high movement as quantified by mean DVARS (Supplementary Fig. 3). See the relation between cluster assignment and the Yeo 17 Network solution in Supplementary Appendix 1.

### Relationship of Lifespan Encoding Clusters with Cognitive Function

**Figure 4:**
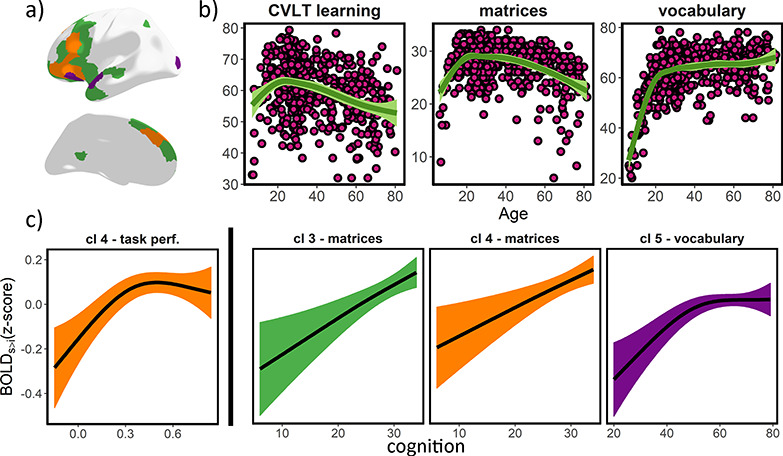
Episodic encoding activity—cognitive function relationships. (*a*) Topography for cluster 3, 4, 5 (green, orange, purple; left hemisphere only). (*b*) Lifespan trajectories of CVLT learning, matrices, and vocabulary scores. (*c*) Significant (FDR-corrected) relationships between cluster activity and cognitive function, controlling for age and sex. *x*-axis represents test scores and *y*-axis represents (source-v-item) encoding activity. Ribbons represent 95% confidence intervals. Note that activity (*y*-axis) values are derived from a PCA (within cluster), and thus demeaned. For clusters 3–5, higher values represent stronger positive memory effects (see Fig. 2). BOLD_S > i_ = Subsequent source versus item memory fMRI contrast.

We next tested whether variations of activity in the encoding clusters related to interindividual differences in core cognitive functions, as indexed by Matrices Reasoning and Vocabulary scores (Wechsler 1999). Further, we tested the relationship between cluster activity and memory performance as indexed both by task performance in the fMRI task as well as by an external verbal recall task (CVLT learning) (Delis 2000). CVLT learning, Matrices Reasoning, and Vocabulary scores were related to memory performance in the task (both adjusted and unadjusted for age). See Supplementary Results and Supplementary Figure 5. We ran parallel GAM models with age and the cognitive tests as smoothing terms, principal component analysis (PCA)-based cluster activity as outcome, and sex as a covariate. See Figure 4 for a visual representation of the relationship between BOLD activity and cognitive function. Activity in cluster 4 was significantly associated with better performance on the fMRI task (*F* = 7.8, pFDR [*n* = 24] = 0.006, edf = 2.2) and matrix scores (*F* = 10.0, pFDR = 0.04, edf = 1.1) while CVLT learning scores were close to significance (*F* = 4.1, pFDR = 0.05, edf = 2.2). Activity in clusters 5 and 3 was associated with better vocabulary and matrix scores), respectively (*F* = 6.6, pFDR = 0.01, edf = 2.7; *F* = 11.4, pFDR = 0.03, edf = 1.3. The remaining comparisons did not pass the significance threshold. Note that the relationship with vocabulary scores in cluster 5 flattens with higher cognitive performance suggesting that the observed non-linear association is mostly driven by the younger participants. See complete stats in Supplementary Table 2. The results indicate that activity in developmentally sensitive clusters is linked to performance in established core functions known to drive cognitive change throughout the lifespan.

### Relationship of Lifespan Encoding Clusters with Large-scale Modes of GM Variation

Next, we assessed whether interindividual differences in activity in developmentally sensitive clusters were associated with core features of structural brain variability throughout the lifespan. We obtained modes of GM variation based on cortical thickness, cortical area and, VBM-based volume. As described in Douaud et al. (2014), we identified two GM components that showed a strong relationship with age. The first, IC_GM_1, represented a dominant whole-brain mode of variation, explaining ∼25% of the structural variance across individuals. This component showed a monotonic decrease in GM across the lifespan. Age explained ∼86% of the IC_GM_1 variance as assessed post-hoc with GAM. The second, IC_GM_2, explained ∼5% of the total GM variance and loaded heavily on prefrontal and parietal heteromodal areas. IC_GM_2 exhibited an inverse U-shape trajectory with age, which explained 46% of the component’s variance. See additional details in Supplementary Methods, Supplementary Results, and Supplementary Figures 6 and 7.

**Figure 5:**
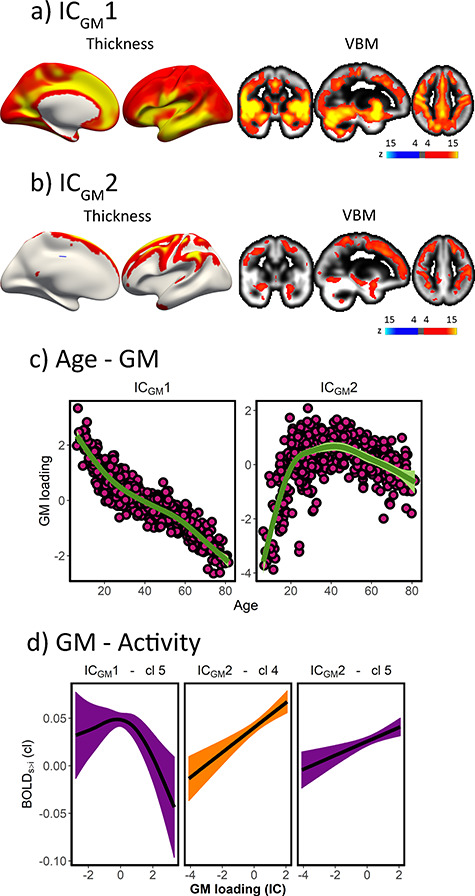
Activity—GM variation. (*a*, *b*) Modes of GM variation with practical (*r*^2^ > 0.15) age significance. Area loadings not displayed; see Supplementary Fig. 7. (*c*) Relationship between age and modes of GM variation. (*d*) Relationship between GM variation and cluster activity (sex, age-corrected). Only significant relationships after FDR-correction are shown. *x*-axis represents GM loadings and *y*-axis represents activity. Ribbons represent 95% confidence intervals. Note that activity (*y*-axis) values are derived from a PCA and thus centered to 0. For clusters 3–5, higher values represent stronger positive memory effects. See Figure 2.

GAM analysis—using age and GM variation as smoothing terms and sex as covariate—revealed that interindividual differences in GM captured by IC_GM_2 related to higher encoding activity in clusters 4 and 5 (*F* = 11.3, pFDR [*n* = 12] = 0.01, edf = 1; *F* = 23.9, pFDR < 0.001, edf = 1, respectively). IC_GM_2 network mapped onto areas susceptible to normal and abnormal childhood and adolescent developmental and aging changes (Douaud et al. 2014). In addition, encoding activity in cluster 5 was associated with GM loadings in IC_GM_1 (*F* = 5.7, pFDR = 0.01, edf = 2.5) (Fig. 5). See full stats in Supplementary Table 3. Note that during child development, the relationship between GM indices such as cortical thinning and cognition is typically negative (Squeglia et al. 2013), which can explain the negative relationship between IC_GM_1 variation and activity, which exists only for high GM loads. Thus, the results suggest that cluster activity is constrained and supported by the development and decay of large modes of GM variation throughout the lifespan.

### Topological Relationship of Lifespan Partitions with Functional and Evolutionary Hierarchies

**Figure 6:**
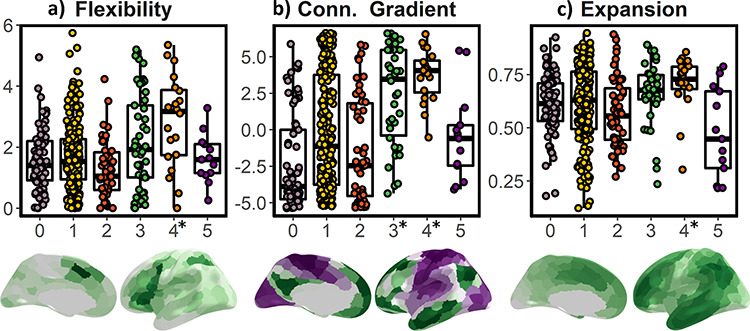
Topological relationship between encoding clusters and functional and evolutionary hierarchy. (*a*) Flexibility (Yeo et al. 2015), (*b*) specialization determined by the principal gradient of functional connectivity (Margulies et al. 2016), and (*c*) macaque-human expansion (Van Essen and Dierker 2007; Hill et al. 2010). Y-label represents the number of recruited components, distance (mm), and normalized (0–1) expansion, respectively. Conn. Gradient = principal gradient of functional connectivity. *Denotes significance after permutation testing.

Finally, we tested whether the lifespan trajectories of encoding activity were embedded in fundamental aspects of brain organization as indexed by flexibility, the principal gradient of functional connectivity, and cortical expansion through evolution. Flexibility indexes the degree to which a region participates in multiple cognitive components, likely by binding and integrating specialized brain networks (Yeo et al. 2015). The principal gradient of functional connectivity represents an overarching organization of large-scale connectivity that reflects a functional hierarchy from perception/action (in sensorimotor areas) to abstract cognitive functions (in the default mode network) (Margulies et al. 2016). The expansion index reflects the degree to which a region has grown in size between macaque and humans (Van Essen and Dierker 2007; Hill et al. 2010). These three measures reflect different fundamental aspects of brain organization in which higher values reflect diminished constraints of sensory and structural input and increased capacity to support a wider array of different tasks such as higher-order cognition (Buckner and Krienen 2013; Sneve et al. 2019; Baum et al. 2020). Figure 6 presents the topological relationship between clusters—based on lifespan trajectories of encoding function—and the functional and evolutionary hierarchical maps. Results showed that cluster 4 encompassed regions characterized by high flexibility (pFDR [*n* = 18] = 0.002), high macaque to human expansion (pFDR = 0.04), and aligned at the apex of the functional connectivity hierarchy (pFDR < 0.001) while cluster 3 was characterized by regions aligned at the apex of the functional connectivity hierarchy (pFDR < 0.001). See complete stats in Supplementary Table 4.

## Discussion

The results suggest that episodic encoding activity exhibits a continuity from childhood to older age, supported by age-sensitive features of brain structure and general cognitive functions. Existing evidence suggests that differences in late-life general cognitive function and brain structure are shaped by early-life influences (Karama et al. 2014; Walhovd et al. 2016). Here we show that this principle also applies to the brain activity underlying episodic encoding success and that some episodic memory trajectories are also determined by fundamental aspects of brain organization such as cognitive flexibility, functional connectivity, and evolutionary cortical expansion. Critically, we did not find evidence of age-specific profiles of activity emerging at middle or older age. Rather, memory encoding trajectories were always influenced by developmental profiles. While some cortical regions showed higher encoding-related activity in older age, they invariably corresponded to monotone increments originating in childhood, suggesting that these patterns did not reflect compensatory responses or dedifferentiation processes appearing at old age. Thus, the results suggest that episodic memory encoding is related to fundamental brain characteristics in much the same way as general cognitive function, and that successful episodic memory encoding in higher age should be understood in a lifespan perspective. The specific results are discussed below.

### Identification of Canonical Lifespan Trajectories of Episodic Encoding Activity

In the present study, the brain was parcellated based solely on the shape of the lifespan trajectories of encoding activity. This novel, data-driven approach revealed several clusters characterized by unique lifespan trajectories that mapped to well-characterized functional and evolutionary patterns in the brain. This observation is illustrated by the inverted U-shaped trajectory of cluster 4 that mostly included bilateral inferior and superior prefrontal regions. Prefrontal cortex activity is thought to reflect a set of cognitive control operations that support the encoding of discrete memory traces (Simons and Spiers 2003). The inverse U-shape trajectory of encoding success through life was closely aligned with those of fluid cognitive abilities and prefrontal brain structure integrity (Raz 2000; McArdle et al. 2002) and fits well with the proposition that strategic components of episodic memory undergo a protracted maturation in childhood and a pronounced decline in old adulthood (Shing et al. 2010). Indeed, we found that cluster 4 activity corresponded to higher fluid intelligence (matrix reasoning performance) and higher GM loadings in a frontoparietal heteromodal network. Cluster 4 regions map to a network characterized by protracted child and adolescent development, that is, prefrontal cortex, and is characterized by marked changes in neurobiology including myelination, synaptic pruning and dendritic remodeling (Paus et al. 2008). This prefrontal activity captured by cluster 4 may, in part, reflect the maturation and decline of cognitive control components of encoding function, thus representing an important mechanism for both general and domain-specific cognitive change throughout life (Craik and Bialystok 2006).

The cluster 5 trajectory was characterized by increasing episodic encoding activity during childhood and adolescence and a plateau through adulthood. This pattern can only be revealed via a lifespan approach, as age-relationships were limited to childhood/adolescence, and the purely early developmental nature of the cluster would have been concealed without mapping across a wider age-rage. This cluster included parts of the anterior lateral temporal cortex, the temporoparietal junction, and anterior inferior frontal regions. One can speculate that cluster 5 reflects a group of regions that are involved in high-level conceptual processes. The trajectories of this cluster mimic the lifespan trajectories for representational knowledge and map well to a network involved in semantic/conceptual processing (Yeo et al. 2011; Andrews-Hanna et al. 2014; Ngo et al. 2019). Cluster 5 activity may thus be particularly sensitive to the maturation of conceptual processing mechanisms. Higher cluster 5 activity further related to higher vocabulary scores and lower GM loadings of the first component. This GM component reflects, to a large extent, cortical thickness, and has a strong negative correlation with age, due to the steep rate of apparent cortical thinning during development (Amlien et al. 2016).

The maintenance of cluster 5 activity combined with the inverse U-shape trajectory of cluster 4 in prefrontal structures and the lifelong stability of medial temporal lobe structures has implications at both ends of the lifespan (see Supplementary Appendix 1 for age effects in each cortical and subcortical ROI; e.g., the four hippocampal ROIs were unrelated to age [pFDR > 0.05]). Our findings are in agreement with the notion that associative encoding during childhood is more dependent on perceptual and associative systems than semantic knowledge and strategic components (Maril et al. 2011; Ofen and Shing 2013). Reliance on prefrontal-based mechanisms has been proposed to increase during childhood and adolescence (Ofen et al. 2007; Shing et al. 2016) while medial temporal lobe activity remains more stable throughout child development (Güler and Thomas 2012; Shing et al. 2016; c.f. Ghetti et al. 2010). These trajectories of memory function are reminiscent of the structural maturation profile of the hippocampus and the prefrontal cortex (Tamnes et al. 2013). In comparison, young adults benefit from recruiting regions assigned to both clusters 4 and 5. One may speculate that the acquisition of semantic knowledge, accessible through developed cognitive control processes, and the interaction with the medial temporal lobe regions leads to “peak” memory performance (van Kesteren et al. 2010). Older adults exhibited less prefrontal cortex activity associated with later associative memory. These results are compatible with the default-executive hypothesis of aging, which posits that with increasing age cognitive processes rely more strongly on semanticized mechanisms (Spreng and Turner 2019). Semanticitazion of cognition in old adulthood affects multiple domains, including memory (Umanath and Marsh 2014). Encoding processes may be less elaborate and rely more strongly on existing knowledge and schema in older adults but are also less accessible for efficient encoding (Craik and Bialystok 2006). While there is ample evidence for lifespan changes in memory function along these lines (see Ofen and Shing 2013; Umanath and Marsh 2014; Spreng and Turner 2019), our study does not provide direct evidence of engagement in strategies and thus this interpretation is speculative. If so, the results bear similarity with the notion of (age-specific) compensation by selection in which older adults engage in different strategies to achieve the same goal. Yet, rather than being specific to old adults, younger peers seem to actively use and benefit from such strategies. Thus, while the results conform to the brain maintenance view to the extent that preserved cognition in aging relates to maintaining youthful brain structure and function (e.g., Fig. 5*d*; Nyberg et al. 2012), one should recognize the relative contributions the different cognitive systems can have on memory encoding throughout the lifespan.

Lifespan variations of episodic encoding activity are linked to variations in GM integrity—likely capturing changes in myelin and dendritic arbors (Whitaker et al. 2016; Wen et al. 2018; Natu et al. 2019)—and to major mechanisms of cognitive change through the lifespan, namely fluid and crystallized abilities (Craik and Bialystok 2006). Further, regions showing the most marked changes in episodic encoding activity through life are located in parts of the cortex characterized by strong expansion in primate evolution with a function less constrained by brain structure and sensory input, and hence, able to support a wider array of different task configurations (Buckner and Krienen 2013; Yeo et al. 2015; Margulies et al. 2016). Previous work has shown that inter-individual differences in cortical morphometry in hotspot regions of expansion are related to general cognitive function (Fjell et al. 2015), brain development (Hill et al. 2010), aging and Alzheimer’s Disease (Fjell et al. 2015), and brain activity both during rest and task execution (Sneve et al. 2019). The alignment with the different cortical organization maps suggests that the encoding activity in these regions represents cognitive elements that are continuously developing throughout life as well as being either uniquely human or at least disproportionally developed in humans. In return, these features might also confer a region with heightened vulnerability to the effects of age and disease (Mesulam 1998; Fjell et al. 2015).

Two technical issues to consider relate to the clustering pipeline and the effects of motion on the results (e.g., cluster solution). The *k*-medoids algorithm is a data-driven clustering—and thus descriptive—method. Different parcellations or dissimilarity matrices may yield different clustering solutions. Also, *k*-medoids is blind to any predefined lifespan trajectory and forces every ROI into a cluster. As such some ROIs might not fit well within a given cluster, yet it is unadvisable to exclude them based on this basis as it assesses similarity to the cluster, not to a predefined trajectory. In any case, these concerns do not have much influence as the present findings replicate in a half-split analysis (Supplementary Fig. 2). The interpretability of data-driven solutions also needs to be considered. See above for discussion on Clusters 4 and 5. Cluster 3 resembles a frontoparietal top-down attentional network, while cluster 2 mimicks the posterior default-mode network that influences memory encoding either through resource reallocation or by performing internally-directed mnemonic processes (Chai et al. 2014; Amlien et al. 2018). Cluster 1, however, includes both regions associated with binding mechanisms and perceptual processes. This heterogeneity may have obscured specific relationships between more specific sets of regions—for example, those involved in binding mechanisms—and memory function. The ability to separate different systems or networks ultimately depends on the number of partitions and the degree in which the different systems exhibit different lifespan trajectories. For example, clustering the brain into *k* = 8 cluster does (Supplementary Fig. 1) separate cluster 1 into a set of stable regions and another group characterized by a monotonical increase of activity. Whether fine-grained solutions are meaningful greatly depends on cluster stability—and thus on the lifespan stability of the trajectories—requiring bigger samples in the upper and lower ends of the agespan.

Certainly, motion is strongly associated with age and affects BOLD signal although the impact is minor when using fMRI contrasts. Yet, motion correction is a double-edged sword as removing participants with high motion introduces sample bias while covarying motion out is also problematic as motion is related to maturation and decline of brain structure (Geerligs et al. 2017). Supplementary analyses (Supplementary Fig. 3) showed that the clustering solution is stable to the removal of participants with high levels of motion.

The degree of generalization and the lack of age-specific mechanisms requires further discussion. We found no evidence of age-specific patterns of activity in old adulthood as defined by either over-recruitment, recruitment of new brain regions, or negative activity-performance relationships. The findings refer to general patterns of activity and thus do not necessarily preclude the existence of specific mechanisms supporting successful encoding in older age but limit its extent to small subsamples of participants or to processes with an unspecific spatial distribution. For a given area, activity may reflect different supporting mechanisms of memory encoding during different periods in life. This issue remains largely hypothetical and we believe it unlikely given our constrained experimental setup. Finally, the present study uses a single—though much-used—index of neural function during memory encoding as captured by a BOLD signal contrast. These constraints considered, the findings provide support for a principle of lifespan continuity and fail to find evidence of “reactive” age-specific mechanisms in olderage.

We do predict the results will extend to other subsequent memory contrasts able to isolate associative mechanisms of episodic memory such as face-name pair associates. Indeed, these studies have repeatedly shown evidence of decreased lateral prefrontal activity in old adulthood (e.g., Dennis et al. 2008; Miller et al. 2008; Kim and Giovanello 2011) and in childhood/adolescence (e.g., Shing et al. 2016) and relative lifelong stability of medial temporal and perceptual regions (Güler and Thomas 2012; Park et al. 2013; de Chastelaine et al. 2016; Shing et al. 2016). It is somewhat more uncertain whether results will generalize to situations characterized by intentional encoding and increased environmental support. Including environmental support or explicit instruction tends to minimize memory performance differences between age groups—especially in children—(Brehmer et al. 2007; Kirchhoff et al. 2012). This boost in performance seems related to the recruitment of frontoparietal regions (Logan et al. 2002; Kirchhoff et al. 2012). Similarly, the results may partially depend on the incidental/intentional nature of the encoding task and the availability of prior knowledge of the different age groups (Schneider et al. 1993; Wagnon et al. 2019) as it can modulate the lifespan trajectories of cognition and neural recruitment for certain regions (i.e., frontoparietal attentional networks) (Maril et al. 2011; Köster et al. 2017). Recognition-like contrasts tend to show different age trajectories (Wang and Cabeza 2016). Recognition procedures target more easily accessible memory representations but also can capture attentional or effort-based processes—as miss trials often capture unattended stimuli. Finally, we expect replication of trajectories of cluster 5 and cluster 4 across cognitive domains in congruence with theories of lifespan cognition. The incidental, associative, and self-referential nature of the encoding task is particularly suited to map processes relevant for everyday function and reflect vulnerable features of episodic memory in old adulthood. Altogether the relationships between the lifespan trajectories of memory activity and cognition, brain organization, and structural integrity were largely exploratory and deserve further replication and generalization efforts.

Finally, the present study consists of cross-sectional, correlational data. Longitudinal studies are needed to characterize intraindividual trajectories of function, reveal lead–lag relationships, and uncover specific genetic and environmental influences on memory function trajectories. Ultimately, only longitudinal data will be capable of revealing the functional determinants of cognitive change throughout life (Raz and Lindenberger 2011).

## Conclusion

The study provides support in favor of stable functional foundations of episodic memory through life, from childhood to older age, instead of qualitatively different, age-specific, mechanisms. Variations in episodic memory were related to fundamental features of brain structure and cognition that characterized development and aging. Lifespan approaches provide a comprehensive framework to better understand brain and cognition in different life periods. We thus conclude that understanding memory vulnerability in older age requires a life-long comprehensive framework that considers normative cognitive, structural, and functional aspects of memory function throughout the lifespan.

## Notes

*Conflict of Interest:* None declared.

## Funding

The Department of Psychology, University of Oslo (to K.B.W., A.M.F.); the Norwegian Research Council (to K.B.W., A.M.F.); the European Research Council’s Starting Grant scheme under grant agreements 283634, 725025 (to A.M.F.) and 313440 (to K.B.W.).

## Supplementary Material

Appendix_S1_bhaa348

SI_mn_ClusterLifespan_bhaa348
