# Supplementary material for: The Functional Foundations of Episodic Memory Remain Stable Throughout the Lifespan
Source: Cereb Cortex. 2020 Nov 30;31(4):2098–110. doi: 10.1093/cercor/bhaa348 (PMC7945016; doi:10.1093/cercor/bhaa348)
Supplement: SI_mn_ClusterLifespan_bhaa348 [file si_mn_clusterlifespan_bhaa348.pdf]

Supplementary information: The functional foundations of episodic memory remain stable throughout the lifespan

Vidal-Piñeiro, D. PhD[1]*, Sneve, MH. PhD[1], Amlien, IK. PhD[1], Grydeland, H. PhD[1], Mowinckel, AM. PhD[1], Roe, J.[1], Sørensen, Ø., PhD[1], Nyberg, LH. PhD[2], Walhovd, KB. PhD[1,3], Fjell, AM. PhD[1,3]

[1]Centre for Lifespan Changes in Brain and Cognition, Department of Psychology, University of Oslo, Oslo, Norway.

[2]Umeå Centre for Functional Brain Imaging, Umeå, Sweden; Department of Integrative Medical Biology, Physiology Section and Department of Radiation Sciences, Diagnostic Radiology, Umeå University, Umeå, Sweden.

[3]Department of radiology and nuclear medicine, Oslo University Hospital, Oslo, Norway

Corresponding author:

Didac Vidal Piñeiro

Department of Psychology, Pb. 1094 Blindern

Oslo, Norway, 0317

d.v.pineiro@psykologi.uio.no

Tel: (+47) -22845061

Supplementary Methods

Participants

The final sample included 540 individuals (females = 366, age = 39.1 [SD = 18.5], age range = 6-82). The participants were recruited from several projects coordinated by the Centre for Lifespan Changes in Brain and Cognition (LCBC, University of Oslo): The Norwegian Mother and Child Cohort Neurocognitive Study (Krogsrud et al. 2014), Neurocognitive Development (Tamnes et al. 2010), Cognition and Plasticity Through the Lifespan (Fjell et al. 2008), Constructive Memory (Sneve et al. 2015), and Neurocognitive plasticity (de Lange et al. 2016). All projects were approved by the Regional Ethical Committee of South Norway. All participants >12 years gave written informed consent, all participants <12 years gave oral informed consent and, for all participants <18 years, written informed consent was obtained from their legal guardians. All participants were screened through health and neuropsychological interviews. Initial exclusion criteria included neurologic or psychiatric disorders, chronic illness, premature birth, learning disabilities, left-handedness or, current use of medicines known to affect nervous system functioning. Participants were further excluded based on the following neuropsychological criteria: score <26 on the Mini-Mental State Examination (MMSE) (Folstein et al. 1975), score of ≥21 in the Beck Depression Inventory (BDI) (Beck and Steer 1987), score <85 on the Wechsler Abbreviated Scale of Intelligence (Wechsler 1999), and a T-score of ≤30 on the California Verbal Learning Test II—Alternative Version (CVLT II) (Delis 2000) immediate delay and long delay. Finally, participants' data were discarded due to technical errors, faulty acquisitions or a low number of trials in a condition of interest (< 6 trials; n = 14).

Experimental design and behavioral analysis

This section describes in detail the experimental design. See **Fig. 1a** for a visual illustration. The stimulus material consisted of 300 black and white line drawings depicting everyday objects and items. The experiment consisted of an incidental encoding task and a surprise test, after ≈ 90 minutes, both

inside the scanner. Only fMRI data during the encoding phase has been used in the present study. The encoding and the retrieval tasks consisted of two and four runs, respectively, that included 50 trials each. All runs started and ended with an 11 s baseline recording period in which a central fixation cross was present. An additional baseline period was also presented once in the middle of each run. In the encoding runs, a trial started with a prerecorded female voice asking through the participant's headphones, either "Can you eat it?" or "Can you lift it?" (in Norwegian). Each question was asked 25 times in each run in a pseudorandomized order. One second after the question onset, a picture of an item appeared on the screen (≈ 10 visual degrees in diameter) together with a response indicator that instructed the participant which button to press to respond "Yes" (the object can be eaten/lifted) or "No" (the object cannot be eaten/lifted). Button-response mapping was counterbalanced across participants. The subject had 2 s to produce a response before the object was replaced by a central fixation cross which remained on the screen throughout the intertrial interval (ITI), that lasted between 1-7 s (exponential distribution over four discrete ITIs; mean duration = 2.98 [SD 2.49] s). Despite the participants' response-dependent nature of subsequent memory designs, the design efficiency – i.e. the distribution of ITIs in each encoding run - was tentatively optimized to ensure sufficient complexity in the recorded BOLD time series (http://surfer.nmr.mgh.harvard.edu/optseq/).

Participants were asked to perform a surprise memory test after ≈ 90 minutes of the last encoding trial. Test trials started with a recorded female voice asking the following (Q1): "Have you seen this item before". Then, a picture of an item appeared on the screen, and the participant was instructed to indicate *Yes* ("I saw the item during the encoding phase") or *No* ("I did not see the item during the encoding phase") with a button press. In each run, 25 old and 25 new items were presented in a pseudorandomized order. Each object stayed on the screen for 2 s; if the participant responded that the item was new or did not respond, the trial ended. If the participant remembered seeing the item (pressed *Yes*), a new question followed (Q2): "Can you remember what you were supposed to do with the item?". A *No* response ended the trial, whereas a *Yes* response, indicating that the participant also

remembered the action associated with the item during the encoding, was followed by a final two-alternative forced-choice question (Q3): “Were you supposed to eat it or lift it?”. Here, the participant had to choose between the two actions “Eat” or “Lift” associated with the item encoding (“I imaged eating/lifting the item during the encoding phase”). The second question was included to discourage guessing behavior on the source memory question (Q3). The participants were verbally instructed minutes before both experimental tasks and did not go through any practice session before entering the scanner.

We computed additional behavioral measures in addition to source memory (Yes response to Q1 and Q2 and correct response to Q3); item memory (correct Yes response to Q1 and either a No response to Q2, or incorrect response to Q3) and miss (incorrect No response to Q1) – which are already specified in the main text (see *Experimental design and behavioral analysis* section). The additional measures corresponded to recognition hits (correct Yes response to Q1, regardless of response to Q2 and 3) and incorrect source judgments (incorrect eat/lift response to Q3). New items were classified either as correct rejections or false alarms. Memory performance in the task was assessed with a corrected source memory performance index (correct answers to Q3 - incorrect answers to Q3). This correction tentatively accounts for processes such as false memories, threshold criteria in Q2 or guessing behavior that affects the raw estimates of source memory performance (Vidal-Piñeiro et al. 2017, 2018). fMRI conditions during encoding were modeled based on the behavioral response during the test phase.

fMRI preprocessing

fMRI data were processed using the “fMRIPrep” preprocessing pipeline(Esteban et al. 2019). Next, we detail the preprocessing steps based on the document generated by the fMRIPrep pipeline (v. 1.2.5). fMRIPrep is based on Nipype (v. 1.1.6) (Gorgolewski et al. 2011) while many of its internal operations use Nilearn 0.5.0 (Abraham et al. 2014).

Anatomical data preprocessing: The T1w image was corrected for intensity non-uniformity (INU) using N4BiasFieldCorrection (Tustison et al. 2010) (ANTs v. 2.2.0), and used as T1w-reference throughout the workflow. The T1w-reference was then skull-stripped using antsBrainExtraction.sh (ANTs v. 2.2.0), using OASIS as target template. Brain surfaces were reconstructed using recon-all (FreeSurfer v. 6.0.1) (Dale et al. 1999a), and the brain mask estimated previously was refined with a custom variation of the method to reconcile ANTs-derived and FreeSurfer-derived segmentation of the cortical gray matter (GM) of Mindboggle (Klein et al. 2017). Spatial normalization to the ICBM 152 Nonlinear Asymmetrical template version 2009c (Fonov et al. 2009) was performed through nonlinear registration with antsRegistration (ANTs v. 2.2.0) (Avants et al. 2008), using brain-extracted versions of both T1w volume and template. Brain tissue segmentation of cerebrospinal fluid (CSF), white-matter (WM) and GM was performed on the brain-extracted T1w using fast (FSL v. 5.0.9) (Zhang et al. 2001).

Functional data preprocessing: For each BOLD run, the following preprocessing was performed. First, a reference volume and its skull-stripped version were generated using a custom methodology of fMRIPrep. A deformation field to correct for susceptibility distortions was estimated based on a field map that was co-registered to the BOLD reference, using a custom workflow of fMRIPrep derived from D. Greve's epidewarp.fsl script and further improvements of HCP Pipelines (Glasser et al. 2013). Based on the estimated susceptibility distortion, an unwarped BOLD reference was calculated for a more accurate co-registration with the anatomical reference. The BOLD reference was then co-registered to the T1w reference using bbregister (FreeSurfer) which implements boundary-based registration (Greve and Fischl 2009). Co-registration was configured with six degrees of freedom. Head-motion parameters with respect to the BOLD reference (transformation matrices, and six corresponding rotation and translation parameters) are estimated before any spatiotemporal filtering using mcflirt (FSL v. 5.0.9) (Jenkinson et al. 2002). BOLD runs were slice-time corrected using 3dTshift from AFNI v.

20160207 (Cox and Hyde 1997). The BOLD time-series (including slice-timing correction when applied) were resampled onto their original, native space by applying a single, composite transform to correct for head-motion and susceptibility distortions. These resampled BOLD time-series will be referred to as preprocessed BOLD in original space, or just preprocessed BOLD. Several confounding time-series were calculated based on the preprocessed BOLD: framewise displacement (FD) was calculated for each functional run, using Nipype's implementation (following the definitions by Power et al. (2014)). Additionally, a set of physiological regressors were extracted to allow for component-based noise correction (CompCor) (Behzadi et al. 2007). Principal components are estimated after high-pass filtering the preprocessed BOLD time-series (using a discrete cosine filter with 128s cut-off). A subcortical mask is obtained by heavily eroding the brain mask, which ensures it does not include cortical GM regions. Six anatomical CompCor (aCompCor) components are then calculated within the intersection of the aforementioned mask and the union of CSF and WM masks calculated in T1w space, after their projection to the native space of each functional run (using the inverse BOLD-to-T1w transformation). The head-motion estimates calculated in the correction step were also placed within the corresponding confounds file. All resamplings can be performed with a single interpolation step by composing all the pertinent transformations (i.e. head-motion transform matrices, susceptibility distortion correction when available, and co-registrations to anatomical and template spaces). Gridded (volumetric) resamplings were performed using antsApplyTransforms (ANTs), configured with Lanczos interpolation to minimize the smoothing effects of other kernels (Lanczos 1964). Non-gridded (surface) resamplings were performed using mri_vol2surf (FreeSurfer).

Linked ICA – Modes of GM variation analysis

To obtain modes of GM variation throughout the lifespan we used a linked Independent Component Analysis (ICA) as implemented in FLICA (http://fsl.fmrib.ox.ac.uk/fsl/fslwiki/FLICA) (Groves et al. 2011, 2012) following the pipeline described by Douaud et al. (2014). The linked-ICA approach provides a data-driven decomposition of the images into spatial components characterizing the intersubject

variability. Independent component approaches are able to model data into a set of – maximally independent; not orthogonal - interpretable features, some of them linked to biophysically plausible underlying mechanisms, which can additionally be linked to external variables such as age. Here, each spatial component represents a mode of variation of GM structure across n = 540 participants. We used three different modalities based on T1w-derived data: cortical thickness and area based on cortical surface reconstructions (Dale et al. 1999a; Fischl et al. 1999a; Fischl and Dale 2000b), and volume from a voxel-based morphometry (VBM) protocol (Good et al. 2001; Douaud et al. 2007).

Imaging processing. Cortical thickness and cortical area maps were obtained through the FreeSurfer v.6.0 cortical reconstruction pipeline (http://surfer.nmr.mgh.harvard.edu/fswiki) (Dale et al. 1999b; Fischl et al. 1999b; Fischl and Dale 2000a). Briefly, the automatized processing pipeline feds on T1w images and includes removal of non-brain tissue, Talairach transformation, intensity correction, tissue and volumetric segmentation, cortical surface reconstruction and cortical parcellation. For each participant, the cortical surfaces were transformed into the *fsaverage5* template and smoothed by 12 mm (FWHM). GM volume maps were obtained through a FSL-VBM optimized protocol (Ashburner and Friston 2000; Smith et al. 2004; Douaud et al. 2007) (fsl.fmrib.ox.ac.uk/fsl/fslwiki/FSLVBM) (FSL v. 6.0.1). The volumes were initially masked by the full brain-segmented volume output from FreeSurfer obtained after nonuniformity correction. The images were then averaged and flipped along the x-axis to create a left-right symmetric, study-specific GM template. Next, the native GM images were non-linearly registered to the study-specific template and "modulated" to correct for local expansion/contraction due to the non-linear component of the spatial transformation. Finally, the modulated grey matter images were smoothed with an isotropic Gaussian kernel (σ = 3 mm; ≈7 FWHM).

Linked ICA. We ran the linked ICA decomposition – as implemented in FLICA (Groves et al. 2011, 2012) - with 70 components as described in Douaud et al. (2014). For each independent component, we initially tested the relationship with age using generalized additive models (GAM; *mgcv* package; knots = 10; spline = “cr”; gamma = 2). After FDR-correction using the Benjamini–Yekutieli procedure (Benjamini and Yekutieli 2001) (pFDR), 20 components were significantly related to age (**Supplementary Fig. 6**). Yet, only two reached *practical* significance; that is, explained 15% of the age variance. These two components, thereafter known as $IC_{GM}1$ and $IC_{GM}2$, were selected for further analyses. To assess the relative contribution of each modality in the independent components, we thresholded the weights at $Z > 4$.

Vertexwise analysis across the entire sample

We carried a vertexwise analysis to display the subsequent memory contrast ($BOLD_{S>i}$: source vs. item memory encoding) cortical map for the entire sample across the cortical mantle (**Fig. 1c**). For each participant (n = 540), we transformed each fMRI regressor from the native to the *fsaverage6* template surface and obtained the $BOLD_{S>i}$ contrast by substracting the item to the source condition. Next, we concatenated the individual $BOLD_{S>i}$ maps and carried a one-sample t-test analysis using *mri_glmfit* (FreeSurfer). Statistical significance was considered at $p < .001$ FDR corrected bilaterally as implemented in *mri_fdr*.

Supplementary Results

Cross-Correlation amongst cognitive tests

The different cognitive measures of interest (CVLT learning, Matrix Reasoning, and Vocabulary scores) were significantly cross-correlated both adjusted and unadjusted for age. Adjustment for age was performed by fitting age to the cognitive measures with generalized additive models [GAM] and using the residuals. This procedure is akin to partial correlation but controls for non-linear relations between the age and the different cognitive tests. Most relevantly, task performance was associated with CVLT learning, Matrix Reasoning, and Vocabulary Scores. When age was not controlled for, the relationships were $r = .42$, $r = .46$, and $r = .15$, respectively. After controlling for age, the relationships were lower for CVLT learning and Matrix reasoning ($r = .27$ and $r = .29$) but higher for Vocabulary ($r = .21$) due to the similar/dissimilar lifespan trajectories of the different cognitive tests. See the cross-correlation matrix in **Supplementary Fig. 5**.

Linked ICA – Modes of GM variation analysis

While 20 components showed (pFDR < .05) significance with age (see **Supplementary Fig. 6**), only two of them achieved a practical significance ($IC_{GM}1$ and $IC_{GM}2$; **Fig. 5**), which were consequently selected for further analysis. Both $IC_{GM}1$ and $IC_{GM}2$ replicate, to a great degree, the previous findings of Douaud et al. (2014). The $IC_{GM}1$ showed a global dominant mode of GM variation and exhibited a monotonic decrease across the lifespan. $IC_{GM}1$ was a multimodal component composed of cortical thickness (56%), volume (32%) and, cortical area (11%) information. Spatially, the $IC_{GM}1$ weighted strongly on large regions of the brain, both for the cortical thickness and the volume modalities. Contributions of cortical area information to $IC_{GM}1$ were limited to sensorimotor and occipital cortices (positive contributions in the gyri, negative in the sulci). $IC_{GM}2$ consisted also on a multimodal component determined to a great degree by cortical thickness information (61%) but also by cortical area and volume data (19% each). $IC_{GM}2$ weighted strongly on heteromodal frontoparietal networks in the

cortical thickness and the volume modalities. In addition, medial temporal structures such as the hippocampus also contributed strongly to the component in the volume modality. The spatial pattern for the cortical area modality was relatively weak (thresholded at Z = 4) exhibiting positive effects in small frontoparietal regions and negative effects in lateral temporal and sensorimotor cortices. See representation in **Supplementary Fig. 7**.

Vertexwise analysis across the entire sample

The BOLD correlates of subsequent episodic memory ($BOLD_{S>i}$) across the entire sample exhibited a canonical pattern of positive and negative subsequent memory effects (Kim 2011). Widespread regions of the cortical mantle exhibited positive subsequent memory effects, particularly in the inferior and superior frontal gyrus, the medial temporal lobe, and in regions corresponding to the dorsal and ventral visual stream (including the fusiform, the isthmus cingulate and the superior parietal cortices). The pattern was left-lateralized. Negative subsequent memory effects were mostly constrained to posteromedial and inferior parietal lobe regions, bilaterally. See **Fig. 1c** for a visual representation.

References

Abraham A, Pedregosa F, Eickenberg M, Gervais P, Mueller A, Kossaifi J, Gramfort A, Thirion B, Varoquaux G. 2014. Machine learning for neuroimaging with scikit-learn. Front Neuroinform. 8.

Ashburner J, Friston KJ. 2000. Voxel-based morphometry--the methods. Neuroimage. 11:805–821.

Avants BB, Epstein CL, Grossman M, Gee JC. 2008. Symmetric diffeomorphic image registration with cross-correlation: evaluating automated labeling of elderly and neurodegenerative brain. Med Image Anal. 12:26–41.

Beck A, Steer R. 1987. Beck depression inventary scoring manual. Psychologi. ed New York: Psychological.

Behzadi Y, Restom K, Liau J, Liu TT. 2007. A component based noise correction method (CompCor) for BOLD and perfusion based fMRI. Neuroimage. 37:90–101.

Benjamini Y, Yekutieli D. 2001. The control of the false discovery rate in multiple testing under dependency. Ann Statist. 29:1165–1188.

Cox RW, Hyde JS. 1997. Software tools for analysis and visualization of fMRI data. NMR Biomed. 10:171–178.

Dale AM, Fischl B, Sereno MI. 1999a. Cortical surface-based analysis. I. Segmentation and surface reconstruction. Neuroimage. 9:179–194.

Dale AM, Fischl B, Sereno MI. 1999b. Cortical surface-based analysis. I. Segmentation and surface reconstruction. NeuroImage. 9:179–194.

de Lange A-MG, Bråthen ACS, Grydeland H, Sexton C, Johansen-Berg H, Andersson JLR, Rohani DA, Nyberg L, Fjell AM, Walhovd KB. 2016. White matter integrity as a marker for cognitive plasticity in aging. Neurobiol Aging. 47:74–82.

Delis DC. 2000. California Verbal Learning Test-Second Edition (CVLT-II). San Antonio, TX: Psychological Corporation.

Douaud G, Groves AR, Tamnes CK, Westlye LT, Duff EP, Engvig A, Walhovd KB, James A, Gass A, Monsch AU, Matthews PM, Fjell AM, Smith SM, Johansen-Berg H. 2014. A common brain network links development, aging, and vulnerability to disease. PNAS. 111:17648–17653.

Douaud G, Smith S, Jenkinson M, Behrens T, Johansen-Berg H, Vickers J, James S, Voets N, Watkins K, Matthews PM, James A. 2007. Anatomically related grey and white matter abnormalities in adolescent-onset schizophrenia. Brain. 130:2375–2386.

Esteban O, Markiewicz CJ, Blair RW, Moodie CA, Isik AI, Erramuzpe A, Kent JD, Goncalves M, DuPre E, Snyder M, Oya H, Ghosh SS, Wright J, Durnez J, Poldrack RA, Gorgolewski KJ. 2019. fMRIPrep: a robust preprocessing pipeline for functional MRI. Nat Methods. 16:111–116.

Fischl B, Dale a M. 2000a. Measuring the thickness of the human cerebral cortex from magnetic resonance images. Proceedings of the National Academy of Sciences of the United States of America. 97:11050–11055.

Fischl B, Dale AM. 2000b. Measuring the thickness of the human cerebral cortex from magnetic resonance images. Proc Natl Acad Sci USA. 97:11050–11055.

Fischl B, Sereno MI, Dale AM. 1999a. Cortical surface-based analysis. II: Inflation, flattening, and a surface-based coordinate system. Neuroimage. 9:195–207.

Fischl B, Sereno MI, Dale AM. 1999b. Cortical surface-based analysis. II: Inflation, flattening, and a surface-based coordinate system. NeuroImage. 9:195–207.

Fjell AM, Westlye LT, Greve DN, Fischl B, Benner T, van der Kouwe AJW, Salat D, Bjørnerud A, Due-Tønnessen P, Walhovd KB. 2008. The relationship between diffusion tensor imaging and volumetry as measures of white matter properties. Neuroimage. 42:1654–1668.

Folstein MF, Folstein SE, McHugh PR. 1975. “Mini-mental state”. A practical method for grading the cognitive state of patients for the clinician. J Psychiatr Res. 12:189–198.

Fonov VS, Evans AC, McKinstry RC, Almli CR, Collins DL. 2009. Unbiased nonlinear average age-appropriate brain templates from birth to adulthood. NeuroImage. Supplement 1:S102.

Glasser MF, Sotiropoulos SN, Wilson JA, Coalson TS, Fischl B, Andersson JL, Xu J, Jbabdi S, Webster M, Polimeni JR, Van Essen DC, Jenkinson M, WU-Minn HCP Consortium. 2013. The minimal preprocessing pipelines for the Human Connectome Project. Neuroimage. 80:105–124.

Good CD, Johnsrude IS, Ashburner J, Henson RN, Friston KJ, Frackowiak RS. 2001. A voxel-based morphometric study of ageing in 465 normal adult human brains. Neuroimage. 14:21–36.

Gorgolewski K, Burns CD, Madison C, Clark D, Halchenko YO, Waskom ML, Ghosh SS. 2011. Nipype: a flexible, lightweight and extensible neuroimaging data processing framework in python. Front Neuroinform. 5:13.

Greve DN, Fischl B. 2009. Accurate and robust brain image alignment using boundary-based registration. Neuroimage. 48:63–72.

Groves AR, Beckmann CF, Smith SM, Woolrich MW. 2011. Linked independent component analysis for multimodal data fusion. Neuroimage. 54:2198–2217.

Groves AR, Smith SM, Fjell AM, Tamnes CK, Walhovd KB, Douaud G, Woolrich MW, Westlye LT. 2012. Benefits of multi-modal fusion analysis on a large-scale dataset: life-span patterns of inter-subject variability in cortical morphometry and white matter microstructure. Neuroimage. 63:365–380.

Jenkinson M, Bannister P, Brady M, Smith S. 2002. Improved optimization for the robust and accurate linear registration and motion correction of brain images. Neuroimage. 17:825–841.

Kim H. 2011. Neural activity that predicts subsequent memory and forgetting: a meta-analysis of 74 fMRI studies. Neuroimage. 54:2446–2461.

Klein A, Ghosh SS, Bao FS, Giard J, Häme Y, Stavsky E, Lee N, Rossa B, Reuter M, Chaibub Neto E, Keshavan A. 2017. Mindboggling morphometry of human brains. PLoS Comput Biol. 13:e1005350.

Krogsrud SK, Tamnes CK, Fjell AM, Amlien I, Grydeland H, Sulutvedt U, Due-Tønnessen P, Bjørnerud A, Sølsnes AE, Håberg AK, Skrane J, Walhovd KB. 2014. Development of hippocampal subfield volumes from 4 to 22 years. Hum Brain Mapp. 35:5646–5657.

Lanczos C. 1964. Evaluation of Noisy Data. Journal of the Society for Industrial and Applied Mathematics: Series B, Numerical Analysis. 1:76–85.

Power JD, Mitra A, Laumann TO, Snyder AZ, Schlaggar BL, Petersen SE. 2014. Methods to detect, characterize, and remove motion artifact in resting state fMRI. Neuroimage. 84:320–341.

Smith SM, Jenkinson M, Woolrich MW, Beckmann CF, Behrens TEJ, Johansen-Berg H, Bannister PR, De Luca M, Drobnjak I, Flitney DE, Niazy RK, Saunders J, Vickers J, Zhang Y, De Stefano N, Brady JM, Matthews PM. 2004. Advances in functional and structural MR image analysis and implementation as FSL. Neuroimage. 23 Suppl 1:S208-219.

Sneve MH, Grydeland H, Nyberg L, Bowles B, Amlien IK, Langnes E, Walhovd KB, Fjell AM. 2015. Mechanisms underlying encoding of short-lived versus durable episodic memories. J Neurosci. 35:5202–5212.

Tamnes CK, Østby Y, Walhovd KB, Westlye LT, Due-Tønnessen P, Fjell AM. 2010. Intellectual abilities and white matter microstructure in development: a diffusion tensor imaging study. Hum Brain Mapp. 31:1609–1625.

Tustison NJ, Avants BB, Cook PA, Zheng Y, Egan A, Yushkevich PA, Gee JC. 2010. N4ITK: improved N3 bias correction. IEEE Trans Med Imaging. 29:1310–1320.

Vidal-Piñeiro D, Sneve MH, Nyberg LH, Mowinckel AM, Sederevicius D, Walhovd KB, Fjell AM. 2018. Maintained Frontal Activity Underlies High Memory Function Over 8 Years in Aging. Cereb Cortex.

Vidal-Piñeiro D, Sneve MH, Storsve AB, Roe JM, Walhovd KB, Fjell AM. 2017. Neural correlates of durable memories across the adult lifespan: brain activity at encoding and retrieval. Neurobiol Aging. 60:20–33.

Wechsler T. 1999. Wechsler abbreviated scale of intelligence. San Antonio, TX.

Zhang Y, Brady M, Smith S. 2001. Segmentation of brain MR images through a hidden Markov random field model and the expectation-maximization algorithm. IEEE Trans Med Imaging. 20:45–57.

Supplementary Figures

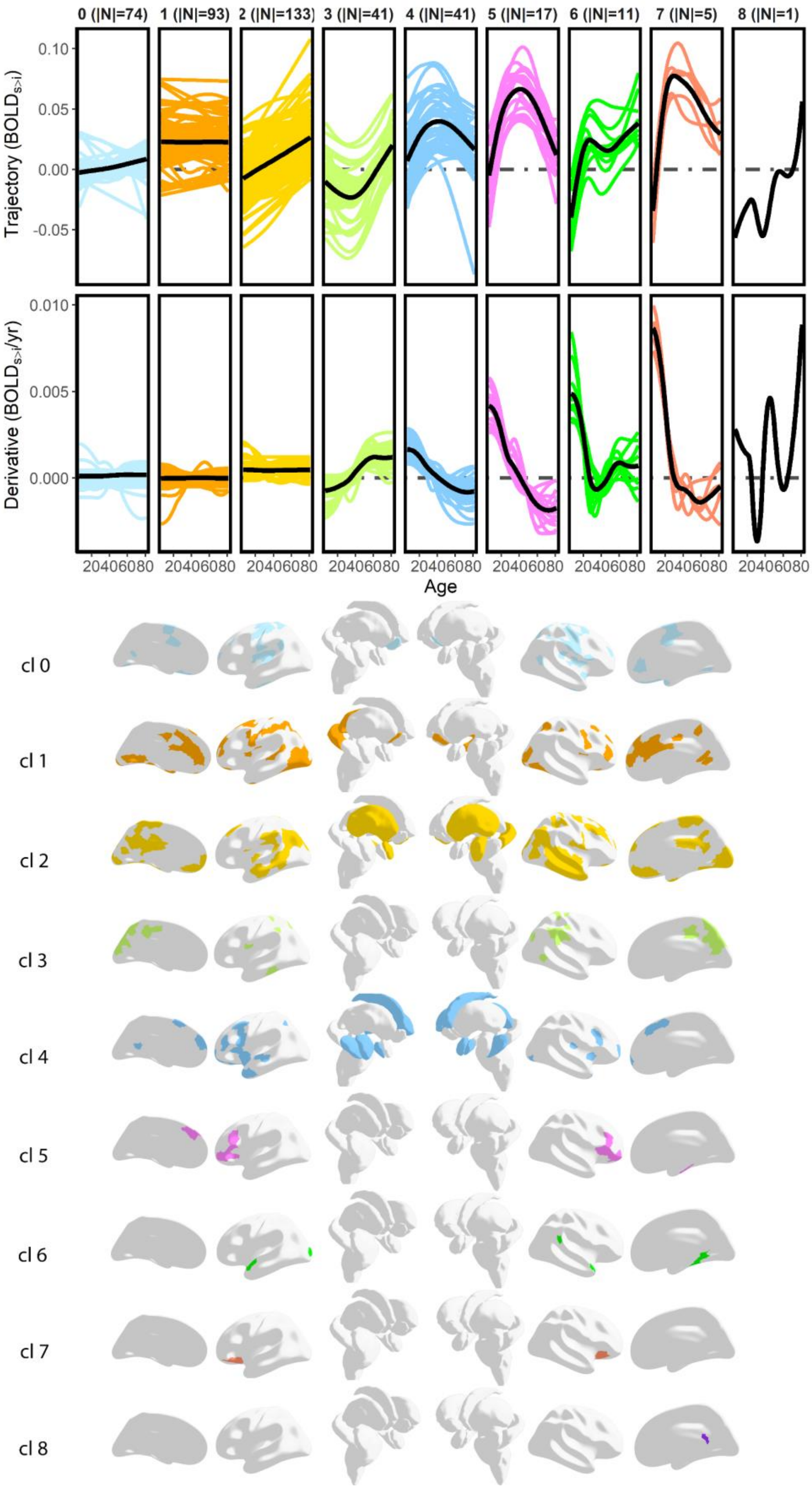

Supplementary Fig. 1. Clustering solution with k = 8 clusters. *Cluster solution based on the derivatives of the lifespan trajectories of encoding activity at a higher dimensionality (k = 8). The k = 8 solution was selected based on a local maxima in the silhouette width coefficient plot (**Fig. 1g**). Upper panel: Lifespan trajectories and the derivatives of encoding activity grouped by cluster. Lower panel: ROI assignment by cluster.* $BOLD_{S>i}$ *= Subsequent source vs. Item memory fMRI contrast.* (|N| = number of ROIs in a cluster). *Compared to the main solution at k = 5 (**Fig. 2**), the k = 8 solution identifies two clusters with monotonical trajectories: one showing lifelong stability and the other characterized by a monotonical increase of activity through the entire lifespan. Further, the k = 8 solution segregates lateral orbitofrontal regions as a separate cluster and isolates an outlier trajectory.*

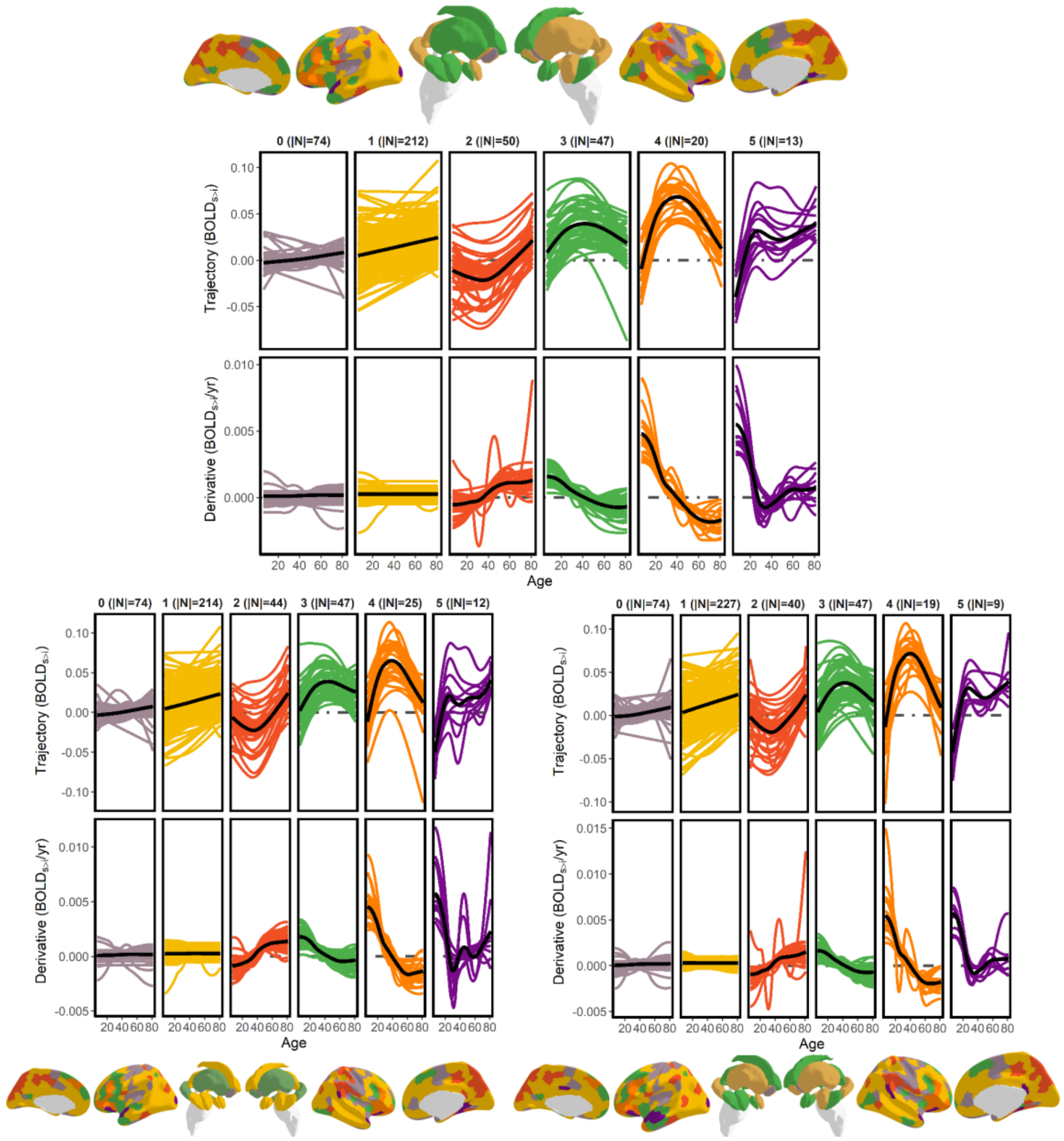

Supplementary Fig. 2. Half-split replication. *Cluster solution based on the derivatives of the lifespan trajectories of encoding activity after half-splitting the sample. The plot shows the lifespan trajectories and the derivatives of encoding activity grouped by cluster (line plots) and ROI assignment by cluster (brain plots). Upper panel: Main results (also shown in* ***Fig.2****). Lower panels: Results from half-split replication. $BOLD_{S>i}$ = Subsequent source vs. Item memory fMRI contrast.* (|N| = number of ROIs in a cluster). The half-split results are comparable to the main results as clusters show a similar lifespan trajectory and spatial distribution. The assignment overlap between the main and the motion-corrected results is 88% and 91%, respectively for the two replications.

.

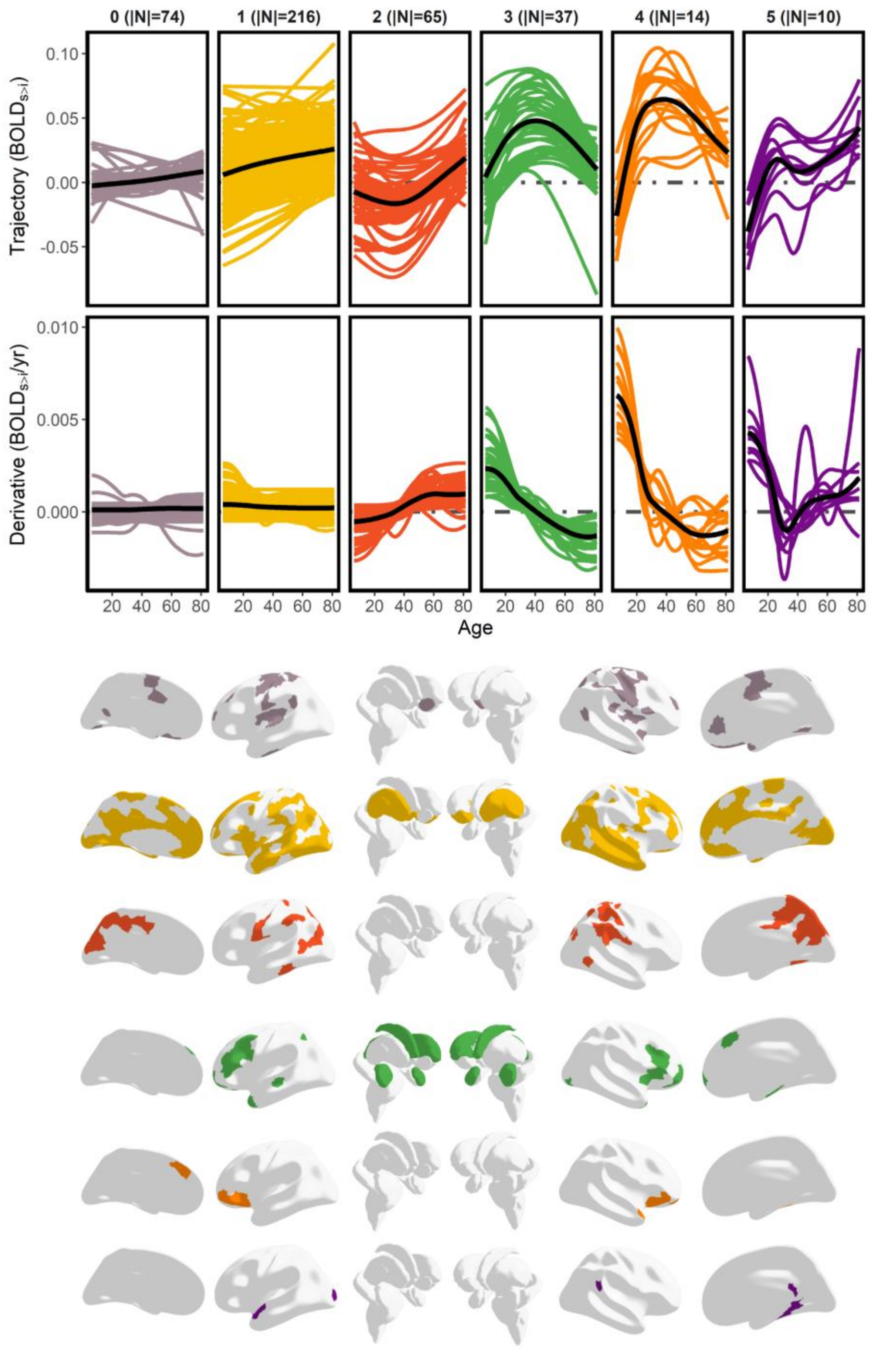

Supplementary Fig. 3. Motion Corrected clustering. *Cluster solution based on the derivatives of the lifespan trajectories of encoding activity after excluding the 10% participants with higher movement as quantified by mean Framewise Displacement (FD). Upper panel: Lifespan trajectories and the derivatives of encoding activity grouped by cluster. Lower panel: ROI assignment by cluster.* $BOLD_{S>i}$ = *Subsequent source vs. Item memory fMRI contrast.* (|N| = number of ROIs in a cluster). The results are comparable to the main results (**Fig. 2**) as clusters show a similar lifespan trajectory and spatial distribution. The assignment overlap between the main and the motion-corrected results is ≥95%.

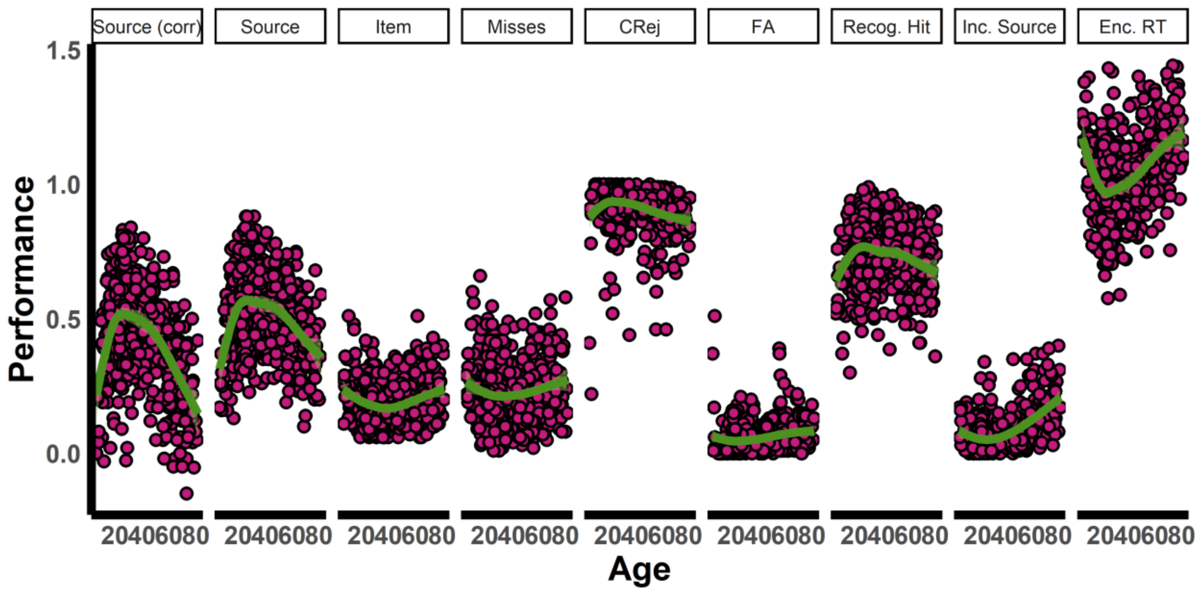

Supplementary Fig. 4. Behavioral measures across the lifespan. *Smoothings are based on the GAM add-on implemented in ggplot2 (formula = y ~ s(x, bs = "cr", k = 8)). Values are proportional. See stats in* **Supplementary *Table 1.*** *Note that the GAM stats* **Supplementary *Table 1*** *are derived from mgcv. Source (corr) = Source hits (corrected); Source = Source hits; CRej = Correct rejections; FA = False alarms; Recog. Hit = Recognition hits; Inc. Source = Incorrect Source Judgments. Enc. RT = Response time during encoding evaluation (in ms).*

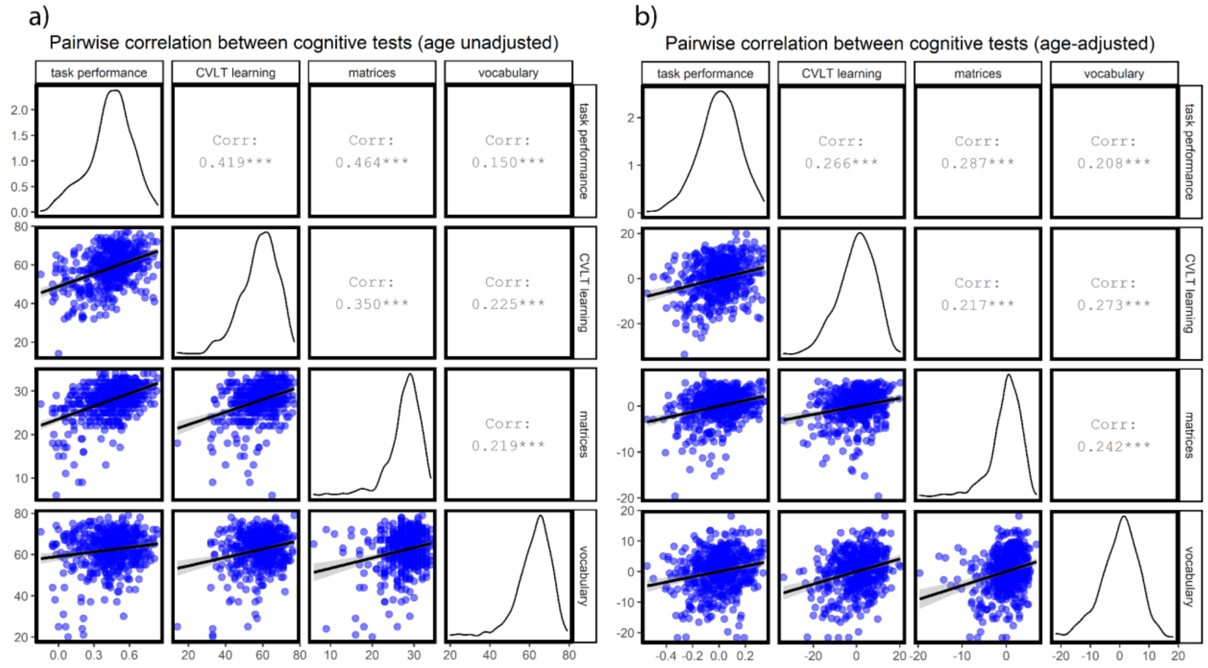

Supplementary Fig. 5. Cross-correlation amongst cognitive tests. *Pairwise correlation between task performance, CVLT learning, matrix reasoning, and vocabulary tests. Both a) unadjusted and b) controlling for age. Scatterplots and linear fitting are shown in the lower triangular matrix. The upper triangular matrix exhibits Pearson's correlation (r) and significance (*** = p < .001). The density distributions for each variable are displayed in the diagonal.*

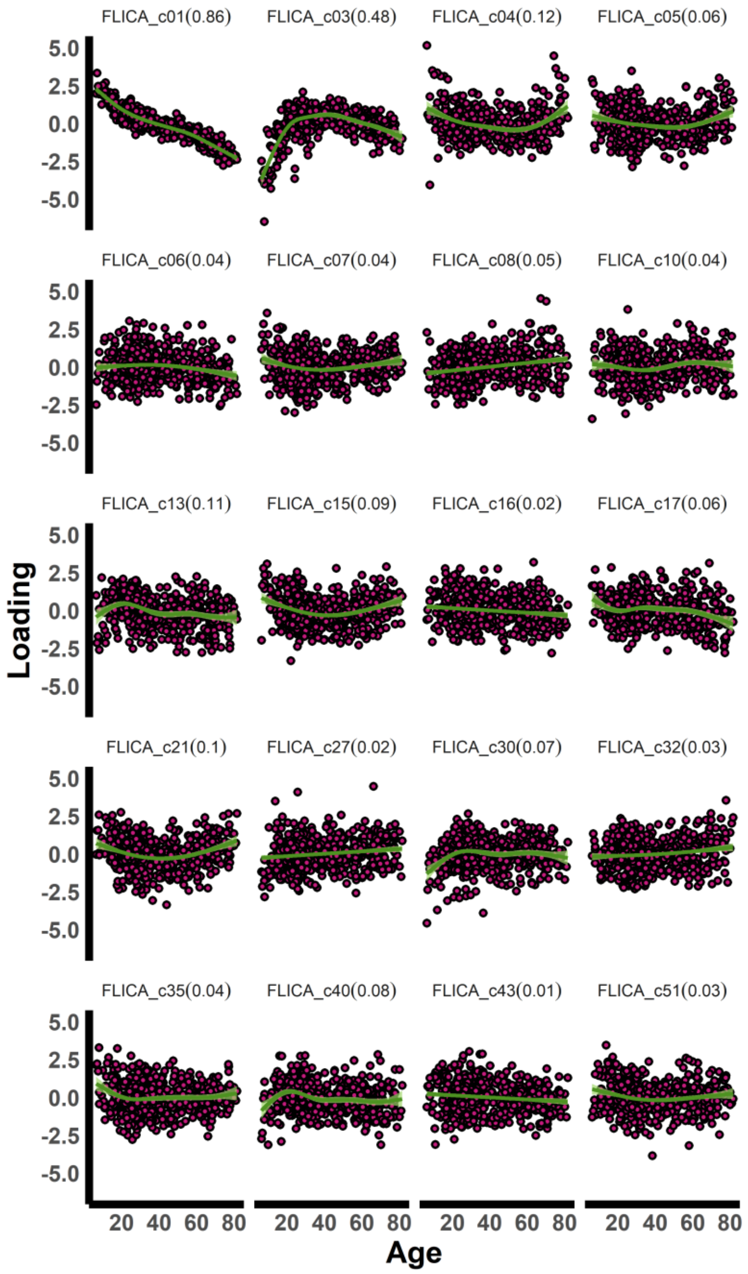

Supplementary Fig. 6. Linked-ICA components associated with age. *Relationship between Independent Components weights and Age. Only components with* pFDR < *.05 are shown. In parenthesis, age variance explained by the component (as assessed with GAM models). Only components «FLICA_01» and «FLICA_03» achieved practical significance and are hereafter referred to as* $IC_{GM}1$ *and* $IC_{GM}2$.

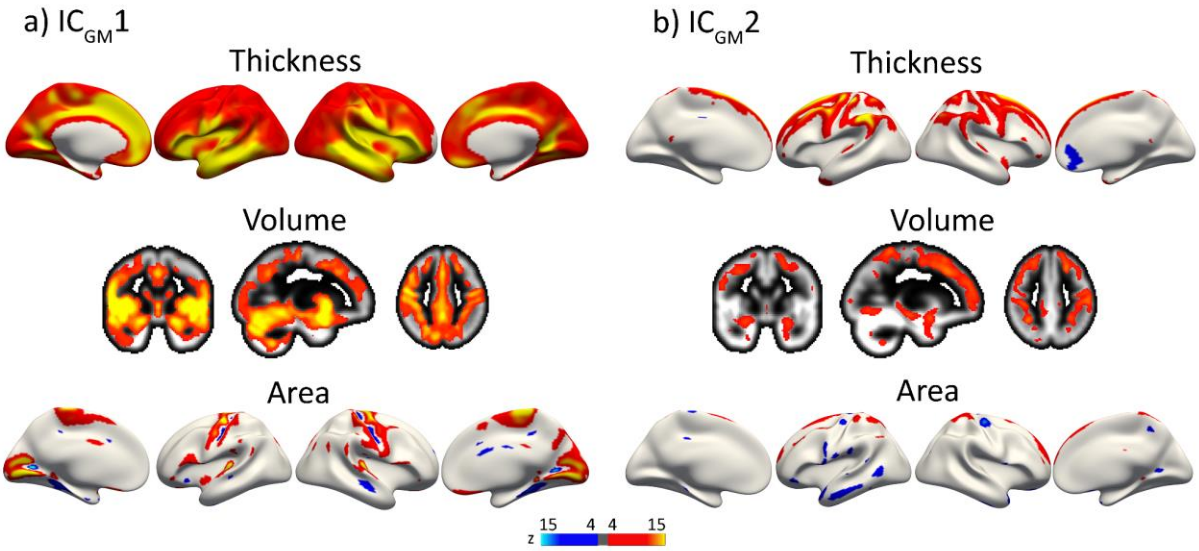

Supplementary Fig. 7. Spatial weight of independent GM components. *a, b) Modes of GM variation with practical (r^2 > .15) age significance. The current Figure expands the representation in **Fig. 5a,b** by including the weights for the cortical area modality and in the right hemisphere for cortical thickness.*

Supplementary Tables

	All	<15	15-20	20-30	30-40	40-50	50-60	60-70	>70	GAM stats
**N Participants**	540	29	35	166	95	59	53	62	41	---
**Source hits (corrected)**	.44(.18)	.33(.20)	.47(.17)	.51(.15)	.51(.14)	.48(.14)	.38(.17)	.31(.18)	.22(.17)	38.7(4.3; <.001)*
**Source hits**	.52(.15)	.41(.17)	.52(.15)	.56(.13)	.56(.13)	.56(.12)	.49(.13)	.45(.14)	.40(.12)	20.4(4.2; <.001)*
**Item**	.19(.08)	.23(.12)	.19(.07)	.18(.07)	.17(.07)	.17(.08)	.20(.08)	.21(.09)	.22(.09)	7(2.8; <.001)*
**Misses**	.23(.11)	.27(.13)	.23(.14)	.21(.11)	.22(.11)	.21(.10)	.24(.12)	.24(.12)	.25(.10)	3.97(2.2; =.06)
**Correct rejections**	.92(.09)	.91(.17)	.94(.07)	.93(.07)	.93(.07)	.92(.07)	.89(.11)	.88(.09)	.87(.07)	11(2.5; <.001)*
**False alarms**	.06(.06)	.06(.11)	.04(.04)	.05(.04)	.05(.04)	.05(.04)	.08(.09)	.08(.05)	.07(.05)	14.7(1.2; <.001)*
**Recognition hits**	.74(.12)	.68(.13)	.74(.14)	.77(.11)	.76(.12)	.75(.11)	.73(.11)	.72(.12)	.70(.11)	7.2(3.1; <.001)*
**Incorrect Source judgments**	.08(.07)	.08(.06)	.05(.04)	.06(.05)	.05(.04)	.08(.05)	.11(.07)	.14(.08)	.18(.09)	64(3.2; <.001)*
**Encoding RT (ms)**	1.03(.15)	1.09(.17)	1.00 (.12)	0.98(.14)	0.99 (.12)	1.01(.13)	1.09(.14)	1.13(.12)	1.18(.14)	26.4(4.0; <.001)*

Supplementary Table 1. Behavioral descriptives. *Mean (SD) of the behavioral measures derived from the fMRI memory task. Descriptives are shown for the entire sample and by age subgroups. Values are proportional. GAM stats indicate F(edf [estimated degrees of freedom]; pFDR). GAM was estimated with knots = 10, bs = "cr", method = "REML" and, gamma = 2. * denotes p < .05.*

	CVLT learning	Matrices	Vocabulary	task perf.
**cl 0**	.62(1,1)	2.56(3.5,.22)	.04(1,1)	.05(1,1)
**cl 1**	1.78(1,.98)	6.57(1,.1	.65(1,1)	.81(1.4,1)
**cl 2**	.05(1.2,1)	.68(1,1)	2.76(1,.59)	4.18(1,.27)
**cl 3**	5.21(1,.19	11.43(1.1,.02)*	5.08(1.8,.06)	3.82(2,.1)
**cl 4**	8.55(1.3,.05)	10.04(1.1,.04)*	4.10(2.2,.08)	7.78(2.2,.01)*
**cl 5**	2.48(1,.66)	7.02(1,.09	6.55(2.1,.01)*	2.80(2.3,.22)

Supplementary Table 2. Relationship between encoding clusters activity and cognition. *GAM stats assessing the relationship between cluster activity and cognition. GAM = F(edf, pFDR). GAM was estimated with knots = 10, bs = "cr", method = "REML" and, gamma = 2. Age (as an additional smoothing term) and Sex were introduced as covariates. * denotes pFDR < .05. Matrices = Matrices Reasoning and Vocabulary = Vocabulary scores from Weschler*(Wechsler 1999). *Task perf = task performance. CVLT learning = California Verbal Learning Test learning score*(Delis 2000).

	$IC_{GM}1$	$IC_{GM}2$
**cl 0**	2.91(1,.55)	2.91(1,1)
**cl 1**	0.39(1,1)	0.39(1,1)
**cl 2**	0.00(1,1)	0.00(1,.55)
**cl 3**	0.20(1,1)	0.20(1,.36)
**cl 4**	1.91(2,.55)	1.91(1,.01)*
**cl 5**	5.70(2.5,.01)*	5.70(1,<.001)*

Supplementary Table 3. Relationship between encoding clusters activity and GM variation. *GAM stats assessing the relationship between cluster activity and GM variation. GAM = F(edf, FDRp). GAM was estimated with knots = 10, bs = "cr", method = "REML" and, gamma = 2. Age (as an additional smoothing term) and Sex were introduced as covariates. * denotes pFDR < .05.*

	Flexibility	Conn. Gradient	Expansion
**cl 0**	1.58(1.00); 1	-2.36(3.22); 1	.61(.15); 1
**cl 1**	1.66(1.08); 1	-0.12(3.93);1	.61(.18); 1
**cl 2**	1.27(0.93); 1	-1.46(3.66);1	.57(.16); 1
**cl 3**	2.16(1.54); .06	2.4(3.40); >.001*	.66(.15); .28
**cl 4**	2.86(1.51); .002*	3.72(1.77); >.001*	.71(.13); .04*
**cl 5**	1.63(0.80); 1	-0.48(3.11); 1	.47(.21); 1

Supplementary Table 4. Topological relationship between encoding clusters and functional and evolutionary hierarchy. *Mean (SD); FDRp values for each functional/evolutionary hierarchy, grouped by cluster. Observations correspond to each of the |N| = 416 ROIs. Conn. Gradient = principal gradient of functional connectivity. * denotes pFDR < .05 using permutation testing.*
